# Simple electrochemical reduction of nitrones to amines†
†Electronic supplementary information (ESI) available: Detailed experimental procedures, characterization data and copies of ^1^H and ^13^C NMR spectra. See DOI: 10.1039/c8sc04337j


**DOI:** 10.1039/c8sc04337j

**Published:** 2018-12-11

**Authors:** Eduardo Rodrigo, Siegfried R. Waldvogel

**Affiliations:** a Institut für Organische Chemie , Johannes-Gutenberg-Universität Mainz , Duesbergweg 10–14 , 55128 Mainz , Germany . Email: waldvogel@uni-mainz.de

## Abstract

The use of electricity allows the reduction of nitrones containing aromatic and heteroaromatic rings to the corresponding amines. The main advantage of this protocol relies on the fact that only electrons are needed, avoiding the use of different chemicals and harsh procedures, something that reinforces some green aspects. The conversion tolerates different moieties and a large variety of functional groups. In addition, this electrolysis can be performed on a simple undivided beaker-type cell with constant current conditions.

## Abstract 2

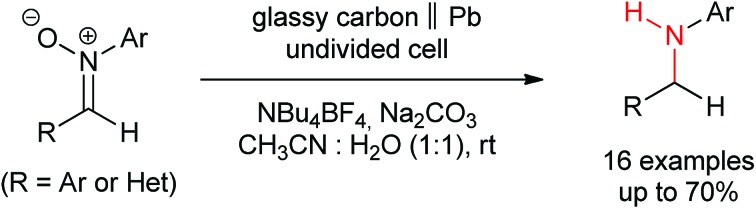
Only electricity is needed for the transformation of nitrones to amines. Such a direct double reduction has not been reported by any sole chemical reagent in a single step process.

## Introduction

The role of nitrones in organic chemistry is very well stablished, fundamentally due to its synthetic versatility.^1^ This is caused principally by the electrophilicity of the double C–N bond, which allows them to act as dipoles in 1,3-dipolar additions in order to afford nitrogen-containing heterocycles, such as isoxazolidines.^2^ Another reaction of interest is their reduction, which has primarily been guided to the synthesis of hydroxylamines.^1^,^3^ However, nitrones can also be reduced and transformed into the corresponding amines. In fact, the reduction of open nitrones to amines needs two independent steps or at least two different reagents for both reductions.^4^ The first step, which allows the formation of the imine through a deoxygenation process, is normally performed using a metal,^4^,^5^ even though there are also examples using different organic reagents.^6^ However, many of these methods are limited by harsh procedures, side reactions or lack of chemoselectivity, whereas the second one to yield the final amine has been often carried out through hydrogenation or the use of hydrides.^7^ Therefore, the reduction of a nitrone into the corresponding amine using one single reagent remains elusive. This is an important aspect, since, for instance, amines are usually synthesized in the pharmaceutical industry through reductive aminations,^8^ and in some cases, anilines are more expensive than the corresponding aryl nitroderivatives.^9^

Recently, the field of organic electrochemistry has experienced a renaissance,^10^ a fact that has permitted the opening of new paths in organic synthesis, as well as the development of novel concepts in the field.^11^ The use of electricity as “single reagent” allows the avoidance of big amounts of oxidizers or reducing agents, something that offers many advantages and benefits from both an ecological and an economic perspective, providing the development of sustainable procedures.

We envisioned that we could carry out the direct reduction of nitrones to amines using electrochemistry. The cleavage of N–O bonds with electricity has been described and it is known for some organic moieties, such as nitrile *N*-oxides or oximes,^12^ whereas there are some electrochemical examples of reductive processes with imines, although guided to the synthesis of diamines.^13^,^14^

**Fig. 1:**
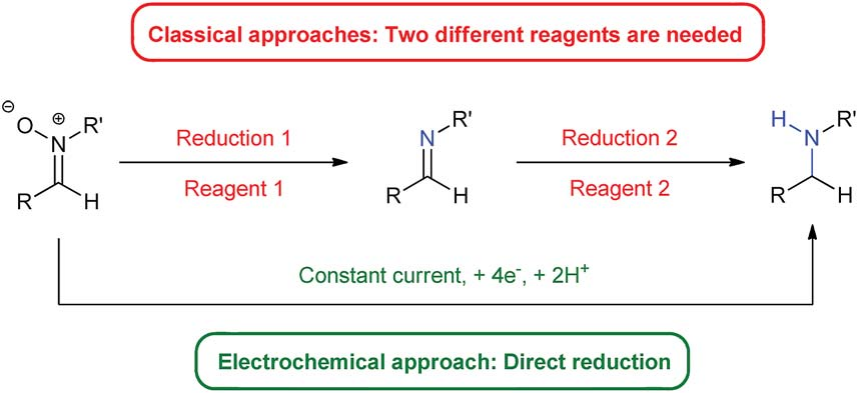
Classical approach *vs.* electrochemical approach.

Herein we present an electro-reduction methodology of nitrones for the synthesis of secondary amines. This approach allows the reduction of experimental steps to the minimum and the avoidance of two different reagents for such a process (Fig. 1), being the first time that this double reduction is performed with one single reagent, something which has not been described yet with any other classical chemical. In addition, the green aspects of the transformation are also important, since only electric current is utilized, something very significant in terms of atom economy and from an ecological point of view.

## Results and discussion

The initial experiments were carried out to adjust the required quantity of charge and to determine the reaction medium. These results are summarized in Table 1.

**Table 1:** Optimization of solvent and additive

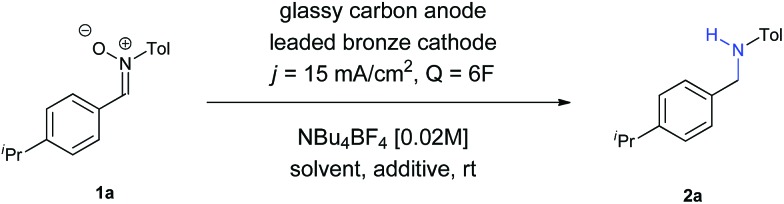				
Entry^*a*^	Solvent	Additive^*c*^	Conv.	Yield (**2a**)^*d*^
1	CH_3_CN^*b*^	—	>95%	11%
2	DMF	—	>95%	10%
3	EtOH	—	94%	24%
4	CH_3_CN/EtOH (1 : 1)	—	>95%	0%
5	CH_3_CN/H_2_O (1 : 1)	—	>95%	29%
6	CH_3_CN/H_2_O (1 : 1)	NEt_3_	>95%	42%
7	CH_3_CN/H_2_O (1 : 1)	NaOH	>95%	38%
8	CH_3_CN/H_2_O (1 : 1)	Na_2_CO_3_	>95%	44%

^*a*^0.15 mmol of nitrone were dissolved in 5 mL of solvent.

^*b*^With *Q* = 4*F*, only 70% was achieved; with *Q* = 5*F*, 83%.

^*c*^0.3 mmol of additive were used.

^*d*^Yields were determined by NMR using 1,3,5-trimethoxybenzene as internal standard.

Firstly, we evaluated the quantity of charge needed for completion of the reaction, in CH_3_CN as solvent, with a glassy carbon anode, a leaded bronze cathode (CuSn_7_Pb_15_) and NBu_4_BF_4_ as supporting electrolyte, at a current density of 15 mA cm^–2^. A minimum theoretical amount of 4*F* was not enough, with some remaining nitrone in the reaction crude. Increasing the charge up to 6*F* yielded the amine, although the yield was very low (Entry 1). The use of DMF did not afford any improvement (Entry 2), while the utilization of a protic solvent such as EtOH (Entry 3) raised the yield. An equimolecular mixture of CH_3_CN and EtOH gave a complex mixture with no formation of the amine (Entry 4), whereas the use of CH_3_CN with another protic solvent such as water was found as optimal (Entry 5). In an electrochemical process for a radical addition of imines to acrylates, the use of triethylamine proved to be efficient to increase the adduct yields.^15^ When using NEt_3_ as additive the yield increased up to 42% (Entry 6). Therefore, other bases such as NaOH and Na_2_CO_3_ were tested (Entries 7 and 8, respectively), being the last one the one who gave better results, raising the yield up to 44%.

At that point, we decided to test how the current density and the electrodes affected the outcome of the reaction (Table 2). To our surprise, an increase of the current density up to 30 mA cm^–2^ led to an increase of the yield (compare Entries 1–3), with larger values decreasing both the conversion and the yield (Entry 4). A plausible explanation for this fact is that, the higher the current density is, the faster the reaction is, and therefore, the intermediates and the radical species involved do not evolve into secondary or undesired products so easily. We have recently observed in our research group in the electrochemical reduction of nitrile oxides,^16^ which present an analogue structure to nitrones, that iminoxyl radicals dimerized to aldazine bis-*N*-oxides, evolving to aldehydes by loss of nitrogen. This issue seems to be eliminated at higher current densities, probably because the further reduction occurs faster when this value is increased. In addition, both the nitrone and the intermediate imine can be sensitive to hydrolysis in the reaction media, so if the reaction time is reduced, this circumstance is also minimized. Finally, it is also well known that when imines are reduced through the intermediate formation of radicals using metals,^17^ photocatalysis^18^ or metal-mediated electrochemistry,^14^ the diamine is obtained, a fact that could also contribute to decrease the final yield of the reaction.

**Table 2:** Optimization of electrolytic conditions

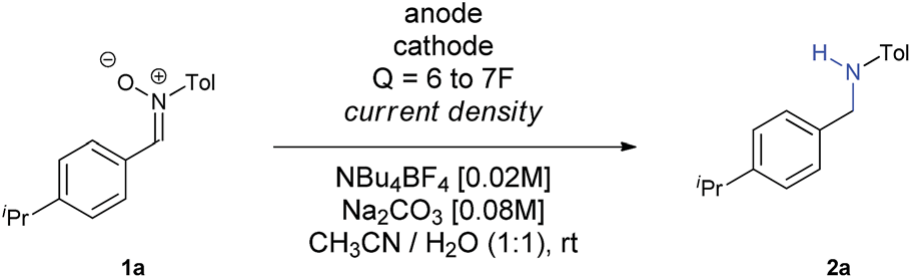						
Entry^*a*^	Anode	Cathode	*Q* (F)	*j* (mA cm^–2^)	Conv.	Yield^*b*^
1	Glassy carbon	Leaded bronze	6	18	>95%	50%
2	Glassy carbon	Leaded bronze	6	24	>95%	51%
3	Glassy carbon	Leaded bronze	6	30	93%	55%
4	Glassy carbon	Leaded bronze	6	45	87%	46%
5	Glassy carbon	Leaded bronze	6.5	30	<95%	56%
6	Glassy carbon	Platinum	6.5	30	80%	19%
7	Glassy carbon	Lead	6.5	30	95%	65%
8	Glassy carbon	Lead	7	30	>95%	67%^*c*^
9	Graphite	Lead	6.5	30	>95%	9%
10	BDD	Lead	6.5	30	>95%	15%

^*a*^0.15 mmol of nitrone were dissolved in 5 mL of solvent.

^*b*^Yields were determined by NMR using 1,3,5-trimethoxybenzene as internal standard.

^*c*^Isolated yield: 60%.

To get full conversion with *j* = 30 mA cm^–2^, 6.5*F* of charge were needed (Entry 5). Other metallic cathodes were tested, being Pb the best one (Entry 7) in comparison to Pt or leaded bronze, though another small increase of charge up to 7*F* was mandatory to get full conversion (Entry 8). Although the counter electrode doesn't always play an important role in electrochemical transformations, in this case the use of other carbon based electrodes as anodes dropped the yield dramatically (Entries 9 and 10), an issue that we observed recently in the electrochemical reduction of nitro derivatives in aqueous conditions.^19^ In this case, its function cannot be considered as secondary, since water seems to provide protons for the reduction and final formation of the amine, while it is also decomposed at the same time at the anode. Furthermore, the basic pH of the reaction media favours this process, since the oxidation potential of water is lower at higher pH values.

**Scheme 1:**
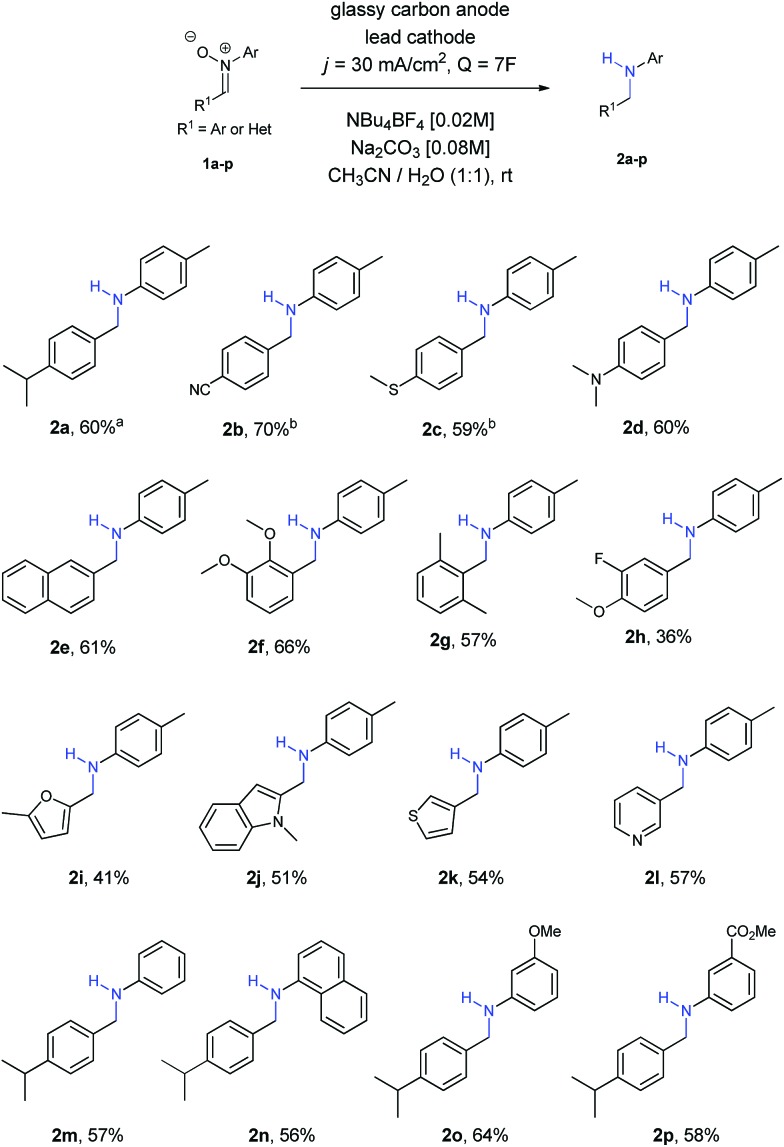
Scope of the reaction. ^*a*^58% yield in a 25 mL beaker type-cell. ^*b*^8*F* of charge were needed.

At this point, we started to evaluate the scope of the reaction. The results are summarized in Scheme 1.

Firstly, we observed that the reaction went smoothly, no matter the nature of the substituent or the substituted position at the ring, with both EWG and EDG, as well as *ortho*, *meta* and *para* positions (amines **2a–2g**). Only in the case of nitrones **1b** and **1c** a small increase of charge (8*F*) was needed to achieve the final amines **2b** and **2c**, owing to solubility reasons. Regarding the use of halogens, only fluorine was tolerated for the reaction, affording amine **2h**, although in lower yields than other functionalities.

Different nitrones with heterocycles were also studied (**1i–1l**), as well as nitrones with different substituents at the ring directly bonded with the nitrogen atom (**1m–1p**). Amines **2i–2l** containing heterocycles and aryl amines **2m–2p** were obtained with moderate to good yields in all the examples.

Aliphatic nitrones were also studied, however their low stability in aqueous solutions^20^ was a major issue and only complex mixtures were observed after isolation. In the case of aliphatic substituents bonded to the nitrogen atom, the reaction was possible, but yields were extremely low and not comparable with the aryl ones.^21^

In order to prove the usefulness of the process, nitrone **1a** was reduced into its corresponding amine **2a** in a 25 mL beaker-type cell, with a very similar yield (58%) under the same reaction conditions. The experimental simplicity of the reaction, in which just a two-electrode arrangement in a beaker-type cell under constant current conditions is used, it also important to be pointed out.

**Scheme 2:**
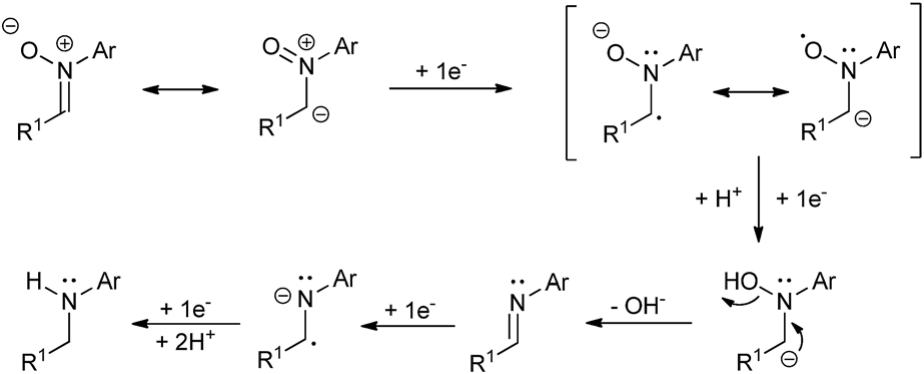
Proposed mechanism for the nitrone reduction.

A plausible mechanism for the described transformation is proposed in Scheme 2. Four electrons are needed for the reduction. The first two electrons yield the imine after cleavage of the N–O bond. The double bond of the imine is subsequently reduced with two more electrons to afford the corresponding amine after protonation. In order to reinforce this mechanistic proposal, we have performed two experiments. In the first one, we have carried out the reduction of **1a** under the optimized conditions and stopped the reaction at *Q* = 3*F*, observing the presence of a small amount of imine (*ca.* 7%) along with **1a** and **2a** in the crude. Secondly, we took commercial *N*-benzylideneaniline and applied *Q* = 0, 1, 2 and 3*F* under the optimized conditions, recording all the NMR spectra. We could see the formation of the corresponding amine from this imine and some byproducts which also explain the loss of yield and Faraday efficiency (see ESI† for more details).

## Conclusions

In summary, we have described an electrochemical method for the reduction of nitrones to amines. A broad scope of aromatic and heteroaromatic amines, containing a large variety of functional groups, was accessible using this methodology. The utilization of electric current allows for the first time the avoidance of two different reagents for this reduction, something that resulted mandatory if this transformation was performed. Furthermore, the simplicity of the method, consisting on an undivided cell under constant current conditions, may open new paths and approaches in terms of amine synthesis.

## Conflicts of interest

There are no conflicts to declare.

## Supplementary Material

Supplementary information

## References

[cit1] Grigoriev I. A., in Nitrile Oxides, Nitrones and Nitronates in Organic Synthesis: Novel Strategies in Synthesis, ed. H. Feuer, Wiley, New Jersey, 2nd edn, 2008, pp. 129–434.

[cit2] Berthet M., Cheviet T., Dujardin G., Parrot I., Martinez J. (2016). Chem. Rev..

[cit3] Bowman W. R. and Marmon R. J., in Comprehensive Organic Functional Group Transformations, ed. A. R. Katrisky, O. Meth-Cohn, C. W. Rees and S. V. Ley, Elsevier Science, Oxford, 1995, vol. 2, p. 344.

[cit4] Radivoy G., Alonso F., Yus M. (2001). Synthesis.

[cit5] Konwar D., Boruah R. C., Sandhu J. S. (1990). Synthesis.

[cit6] Hortmann A. G., Koo J. Y., Yu C. C. (1978). J. Org. Chem..

[cit7] (b) Baxter E. W. and Reitz A. B., in Organic Reactions, ed. Larry E. Overman, John Wiley and Sons, New York, 2001, vol. 59.

[cit8] Warren S. and Wyatt P., in Organic Synthesis: The Disconnection Approach, Wiley-Blackwell, Oxford, 2nd edn, 2008, pp. 53–60.

[cit9] For example, the price of 100 g of *m*-toluidine (99%) in Sigma-Aldrich (November 2018) is 28.60€, while the price of 100 g of *m*-nitrotoluene (99%) in the same company is only 15.90€. For the *ortho* derivatives, the price of 100 g of *o*-toluidine (≥99%) is 41.10€, whereas the price of 100 mL of *o*-nitrotoluene (≥99%) goes down to 23.30€

[cit10] Waldvogel S. R., Janza B. (2014). Angew. Chem., Int. Ed..

[cit11] Yan M., Kawamata Y., Baran P. S. (2017). Chem. Rev..

[cit12] Hammerich O., in Organic Electrochemistry: Revised and Expanded, ed. O. Hammerich and B. Speiser, CRC Press, Boca Raton, 5th edn, 2016, pp. 599–600.

[cit13] Horner L., Skaletz D. H. (1975). Liebigs Ann. Chem..

[cit14] Siu T., Li W., Yudin A. K. (2001). J. Comb. Chem..

[cit15] Shono T., Kise N., Kunimi N., Nomura R. (1991). Chem. Lett..

[cit16] Hartmer M. F., Waldvogel S. R. (2015). Chem. Commun..

[cit17] Tsukinoki T., Nagashima S., Mitoma Y., Tashiro M. (2000). Green Chem..

[cit18] Okamoto S., Ariki R., Tsujioka H., Sudo A. (2017). J. Org. Chem..

[cit19] Rodrigo E., Waldvogel S. R. (2018). Green Chem..

[cit20] Sandler S. R. and Karo W., in Organic functional group preparations, Academic Press, New York, vol. 12-III, 1972, p. 301, and references cited herein.

[cit21] In the case of R = *t*-Bu, the NMR yield was 15%, and for R = Pr, the NMR yield was lower than 10% (1,3,5-trimetoxybenzene was used as internal standard)

