# Supplementary material for: Simple electrochemical reduction of nitrones to amines
Source: Chem Sci. 2018 Dec 11;10(7):2044–7. doi: 10.1039/c8sc04337j (PMC6375361; doi:10.1039/c8sc04337j)

Electronic Supplementary Material (ESI) for Chemical Science.
This journal is © The Royal Society of Chemistry 2018

Electronic Supplementary Information

for

Simple electrochemical reduction of nitrones to amines

Eduardo Rodrigo and Siegfried. R. Waldvogel

Institut für Organische Chemie
Johannes-Gutenberg-Universität Mainz
Duesbergweg 10–14
55128 Mainz (Germany)

Table of contents

Page

1. General information S3

2. General procedure for the electrosynthesis of amines **2** S4

3. Characterization of amines **2** S6

4. Mechanistic studies S12

5. ^{1}H and ^{13}C spectra of amines **2** S15

1. General information

All reagents were used in analytical grades. Solvents were purified by standard methods. For electrochemical reactions, lead and glassy carbon electrodes were used.

Nitrones 1 were synthesized electrochemically, according to the described experimental procedure,[1] except nitrones **1d**, **1h, 1i** and **1p**, which were synthesized using another described procedure.[2]

NMR spectra: ^{1}H NMR and ^{13}C NMR spectra were recorded at 25 °C by using a Bruker Avance III HD 300 (300 MHz, 5 mm BBFO-SmartProbe with z gradient and ATM, SampleXPress 60 sample changer, Analytische Messtechnik, Karlsruhe, Germany). Chemical shifts (*δ*) are reported in parts per million (ppm) relative to traces of $CHCl_3$ (7.26 ppm for ^{1}H NMR and 77.0 ppm for ^{13}C NMR) in the corresponding deuterated solvent.

Mass Spectrometry: Mass spectra and high resolution mass spectra were obtained by using a QTof Ultima 3 (Waters, Milford, Massachusetts) apparatus employing ESI^{+}.

Gas chromatography was performed with a Shimadzu GC-2010 (Shimadzu, Japan) using a HP 5 column (Agilent Technologies, USA; length: 30 m, inner diameter: 0.25 mm, film: 0.25 µm, carrier gas: hydrogen). GC-MS measurements were carried out with a Shimadzu GC-2010 (Shimadzu, Japan) using a Zebron ZB-5MSi column (Phenomenex, USA; length: 30 m, inner diameter: 0.25 mm, film: 0.25 µm, carrier gas: helium) combined with a GCMS-QP2010.

Column chromatography was performed on silica gel 60 M (0.040-0.063 mm, Macherey-Nagel GmbH & Co, Düren, Germany) with a maximum pressure of 1.2 bar. As eluents mixtures of cyclohexane and ethyl acetate were used. Silica gel 60 sheets on aluminum (F254, Merck, Darmstadt, Germany) were used for thin layer chromatography.

Melting points were determined with a Melting Point Apparatus B-545 (Büchi, Flawil, Switzerland) and were uncorrected. The heating rate was 3°C/min.

[1] E. Rodrigo and S. R. Waldvogel, *Green Chem*, 2018, **20**, 2013.
[2] V. Gautheron-Chapoulaud, S. U. Pandya, P. Cividino, G. Masson, S. Py and Y. Vallée, *Synlett*, 2001, **8**, 1281.

2. General procedure for the electrosynthesis of amines 2

Screening cell (beaker-type cell, 5 mL)

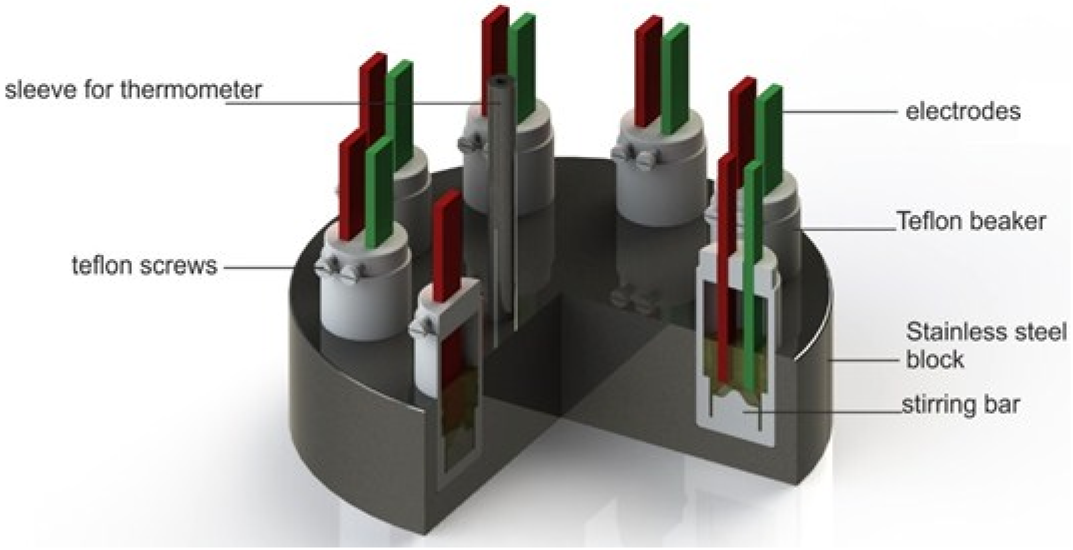

Figure 1. Schematic view of undivided screening cells of 5 mL in a screening arrangement.

A detailed description of this set up was published recently.[3] In addition, the screening set-up is also commercially available as IKA Screenings System, IKA-Werke GmbH & Co. KG, Staufen, Germany. Set-ups with electrodes in a parallel orientation were conducted in a 5 mL teflon cells (Figure 1). Dimensions of glassy carbon and Pb electrodes were 7.0 x 1.0 x 0.3 cm. Using a 5 mL reaction mixture, electrodes immersed 1.65 cm into solution and had upon immersion into the electrolyte an active surface of 1.65 cm^2 (1 cm x 1.65 cm).

Into an undivided beaker-type screening electrolysis cell equipped with a glassy carbon anode and a Pb cathode, CH_3CN (2.5 mL), water (2.5 mL), Na_2CO_3 (42.4 mg, 0.4 mmol), NBu_4BF_4 (32.9 mg, 0.01 mmol), and freshly prepared nitrone **1** (0.15 mmol) were added. A constant current electrolysis with a current density of 30 mA/cm^2 (I = 50 mA) was performed at room temperature. After application of 101 C (7F)[4] the electrolysis was stopped.

The mixture was then transferred into a separation funnel, whereby AcOEt (20 mL) and water (20 mL) were added. The aqueous layer was again extracted with AcOEt (20 mL), the combined organic fractions were washed with brine (20 ml) and dried over $MgSO_4$. The solution was concentrated under reduced pressure and the crude was purified under flash column chromatography to afford the corresponding amines **2**.

---

[3] C. Gütz, B. Klöckner and S. R. Waldvogel, *Org. Process Res. Dev.*, 2016, **20**, 26.
[4] For compounds **1b** and **1c**, 116C (8F) were needed.

Beaker-type cell (25 mL)

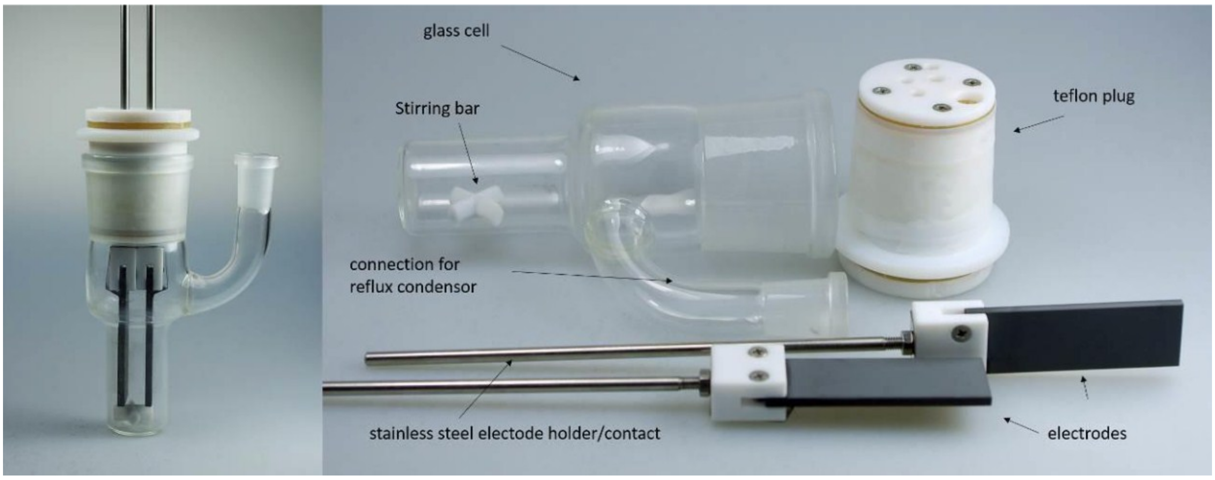

Figure 2. View of undivided Beaker-type cell of 25 mL.

The beaker-type cell (25 mL) consists of a simple glass beaker with or without cooling jacket and closed by a teflon plug. This cap allows precise arrangement of the electrodes. Dimensions of glassy carbon and lead electrodes were 7.0 x 2.0 x 0.2 cm. The electrodes had upon immersion into the electrolyte an active surface of 7 cm^2 (2 x 3.5 cm).

The scale-up of amine **2a** was carried out using this cell.

Into an undivided beaker-type screening electrolysis cell equipped with a glassy carbon anode and a Pb cathode, CH_3CN (12.5 mL), water (12.5 mL), Na_2CO_3 (210 mg, 2 mmol), NBu_4BF_4 (165 mg, 0.05 mmol), and freshly prepared nitrone **1a** (190 mg, 0.75 mmol) were added. A constant current electrolysis with a current density of 30 mA/cm^2 (I = 210 mA) was performed at room temperature. After application of 507 C (7F) the electrolysis was stopped.

The mixture was then transferred into a separation funnel, whereby AcOEt (100 mL) and water (100 mL) were added. The aqueous layer was again extracted with AcOEt (100 mL), the combined organic fractions were washed with brine (100 mL) and dried over $MgSO_4$. The solution was concentrated under reduced pressure and the crude was purified under flash column chromatography under flash column chromatography (cyclohexane/EtOAc = 30:1) to afford the corresponding amine **2a** (104 mg, 58% yield).

3. Characterization of amines

***N*-(4-Isopropylbenzyl)-4-methylaniline (2a)**

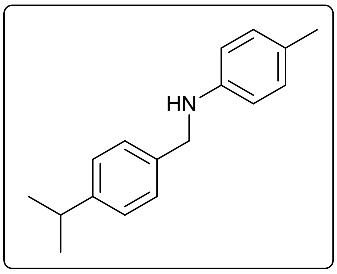

Yellow oil. Purified by flash column chromatography (cyclohexane/EtOAc = 30:1). Yield: 60% (21.5 mg). When the reaction was scaled up (see page S5), yield: 58% (104 mg).

^{1}H NMR ($CDCl_3$, 300 MHz): δ 7.35 (d, *J* = 8.1 Hz, 2H), 7.25 (d, *J* = 8.1 Hz, 2H), 7.04 (d, *J* = 8.0 Hz, 2H), 6.63 (d, *J* = 8.0 Hz, 2H), 4.31 (s, 2H), 2.95 (hep, *J* = 6.9 Hz, 1H), 2.29 (s, 3H), 1.30 (d, *J* = 6.9 Hz, 6H).

^{13}C NMR ($CDCl_3$, 75 MHz): δ 147.92 (C), 145.92 (C), 136.88 (C), 129.76 (CH), 127.67 (CH), 126.79 (C), 126.67 (CH), 113.08 (CH), 48.52 (CH_2), 33.84 (CH), 24.07 (CH_3), 20.44 (CH_3).

HRMS (ESI): calculated for $C_{17}H_{22}N$ $(M + H)^+$: 240.1747; found: 240.1748.

4-((4-Tolylamino)methyl)benzonitrile (2b)

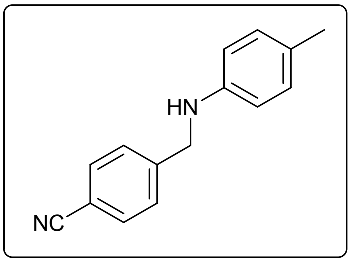

Yellow solid (m.p. = 85-86°C). Purified by flash column chromatography (cyclohexane/EtOAc = 10:1). Yield: 70% (31.2 mg).

^{1}H NMR ($CDCl_3$, 300 MHz): δ 7.63 (d, *J* = 8.3 Hz, 2H), 7.50 (d, *J* = 8.3 Hz, 2H), 7.01 (d, *J* = 8.1 Hz, 2H), 6.58 (d, *J* = 8.1 Hz, 2H), 4.43 (s, 2H), 2.26 (s, 3H).

^{13}C NMR ($CDCl_3$, 75 MHz): δ 145.32 (C), 144.74 (C), 132.42 (CH), 129.87 (CH), 127.80 (CH), 127.70 (C), 118.89 (C), 113.26 (CH), 110.93 (C), 48.29 (CH_2), 20.40 (CH_3).

HRMS (ESI): calculated for $C_{15}H_{15}N_2$ $(M + H)^+$: 223.1230; found: 223.1228.

4-Methyl-*N*-(4-(methylsulfanyl)benzyl)aniline (2c)

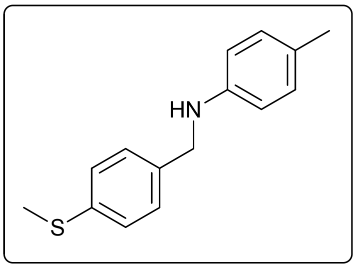

Pale yellow solid (m.p. = 72-74°C). Purified by flash column chromatography (cyclohexane/EtOAc = 20:1). Yield: 59% (28.1 mg).

^{1}H NMR ($CDCl_3$, 300 MHz): δ 7.33 (d, *J* = 8.3 Hz, 2H), 7.25 (d, *J* = 8.3 Hz, 2H), 7.03 (d, *J* = 8.1 Hz, 2H), 6.60 (d, *J* = 8.1 Hz, 2H), 4.30 (s, 2H), 2.51 (s, 3H), 2.28 (s, 3H).

^{13}C NMR ($CDCl_3$, 75 MHz): δ 145.69 (C), 137.07 (C), 136.57 (C), 129.77 (CH), 128.06 (CH),

126.98 (CH), 126.93 (C), 113.12 (CH), 48.24 (CH_2), 20.44 (CH_3), 16.07 (CH_3).

HRMS (ESI): calculated for $C_{15}H_{18}NS$ $(M + H)^+$: 244.1154; found: 244.1156.

***N,N*-Dimethyl-4-((4-tolylamino)methyl)aniline (2d)**

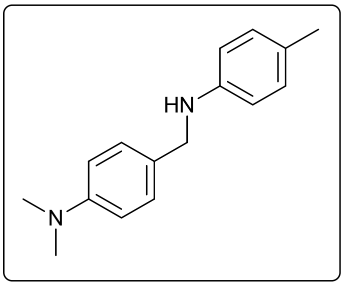

Pale brown solid (m.p. = 98-100°C). Purified by flash column chromatography (cyclohexane/EtOAc = 15:1). Yield: 60% (21.6 mg).

^{1}H NMR ($CDCl_3$, 300 MHz): δ 7.27 (d, *J* = 8.7 Hz, 2H), 7.01 (d, *J* = 8.2 Hz, 2H), 6.75 (d, *J* = 8.7 Hz, 2H), 6.60 (d, *J* = 8.2 Hz, 2H), 4.21 (s, 2H), 2.96 (s, 6H), 2.27 (s, 3H).

^{13}C NMR ($CDCl_3$, 75 MHz): δ 149.97 (C), 146.16 (C), 129.71 (CH), 128.74 (CH), 127.35 (C), 126.55 (C), 113.03 (CH), 112.84 (CH), 48.34 (CH_2), 40.79 (CH_3), 20.44 (CH_3).

HRMS (ESI): calculated for $C_{16}H_{21}N_2$ $(M + H)^+$: 241.1699; found: 241.1700.

4-Methyl-*N*-(naphth-2-ylmethyl)aniline (2e)

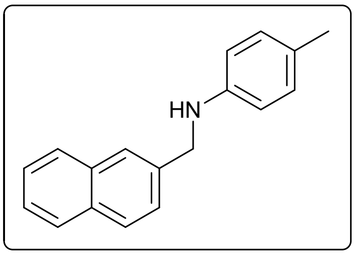

Pale yellow solid (m.p. = 51-53°C). Purified by flash column chromatography (cyclohexane/EtOAc = 6:1). Yield: 61% (22.4 mg).

^{1}H NMR ($CDCl_3$, 300 MHz): δ 7.89-7.81 (m, 4H), 7.56-7.46 (m, 3H), 7.03 (d, *J* = 8.2 Hz, 2H), 6.65 (d, *J* = 8.2 Hz, 2H), 4.51 (s, 2H), 2.28 (s, 3H).

^{13}C NMR ($CDCl_3$, 75 MHz): δ 145.66 (C), 136.99 (C), 133.48 (C), 132.75 (C), 129.79 (CH), 128.34 (CH), 127.77 (CH), 127.70 (CH), 127.09 (C), 126.13 (CH), 125.98 (CH), 125.81 (CH), 125.70 (CH), 113.30 (CH), 49.00 (CH_2), 20.43 (CH_3).

HRMS (ESI): calculated for $C_{18}H_{18}N$ $(M + H)^+$: 248.1434; found: 248.1433.

_N_-(2,3-Dimethoxybenzyl)-4-methylaniline (2f)

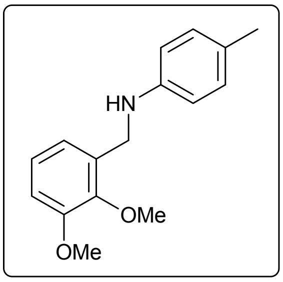

Yellow solid (m.p. = 89-90°C). Purified by flash column chromatography (cyclohexane/EtOAc = 10:1). Yield: 66% (26.2 mg).

^{1}H NMR ($CDCl_3$, 300 MHz): δ 7.07-6.96 (m, 4H), 6.87 (dd, *J* = 7.8 and 1.7 Hz, 1H), 6.62 (d, *J* = 8.3 Hz, 2H), 4.36 (s, 2H), 3.90 (s, 6H), 2.26 (s, 3H).

^{13}C NMR ($CDCl_3$, 75 MHz): δ 152.70 (C), 147.06 (C), 145.84 (C), 133.20 (C), 129.71 (CH), 126.80 (C), 124.09 (CH), 120.97 (CH), 113.33 (CH), 111.50 (CH), 60.80 (CH_3), 55.77 (CH_3), 43.67 (CH_2), 20.43 (CH_3).

HRMS (ESI): calculated for $C_{16}H_{20}NO_2$ $(M + H)^+$: 258.1489; found: 258.1492.

_N_-(2,6-Dimethylbenzyl)-4-methylaniline (2g)

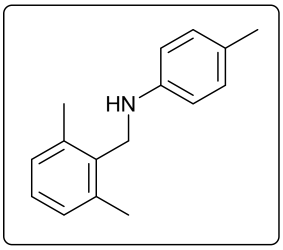

Yellow solid (m.p. = 73-74 °C). Purified by flash column chromatography (cyclohexane/EtOAc = 20:1). Yield: 57% (19.3 mg).

^{1}H NMR ($CDCl_3$, 300 MHz): δ 7.21-7.03 (m, 5H), 6.65 (d, *J* = 8.4 Hz, 2H), 4.24 (s, 2H), 2.42 (s, 6H), 2.30 (s, 3H)

^{13}C NMR ($CDCl_3$, 75 MHz): δ 146.24 (C), 137.62 (C), 135.25 (C), 129.80 (CH), 128.34 (CH), 127.66 (CH), 126.67 (C), 112.72 (CH), 43.04 (CH_2), 20.45 (CH_3), 19.54 (CH_3).

HRMS (ESI): calculated for $C_{16}H_{20}N$ $(M + H)^+$: 226.1590; found: 226.1592.

_N_-(3-Fluoro-4-methoxybenzyl)-4-methylaniline (2h)

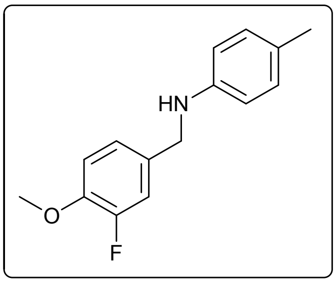

Brown oil (m.p. = 89-90°C). Purified by flash column chromatography (cyclohexane/EtOAc = 30:1). Yield: 36% (13.3 mg).

^{1}H NMR ($CDCl_3$, 300 MHz): δ 7.15-7.04 (m, 2H), 7.00 (d, *J* = 8.1 Hz, 2H), 6.96-6.88 (t, *J* = 8.2 Hz, 1H), 6.58 (d, *J* = 8.1 Hz, 2H), 4.25 (s, 2H), 3.88 (s, 3H), 2.25 (s, 3H).

^{13}C NMR ($CDCl_3$, 75 MHz): δ 154.06 and 150.80 (CF, d, *J* = 247 Hz), 146.80 (C), 146.66 (C), 144.96 (C), 132.35 (C), 129.78 (CH), 127.54 (C), 123.16 (CH), 123.11 (CH), 115.49 (CH), 115.24 (CH), 113.53 (CH), 113.45 (CH), 113.42 (CH), 56.34 (CH_3), 48.08 (CH_2), 20.43 (CH_3).

HRMS (ESI): calculated for $C_{15}H_{17}NFO$ $(M + H)^+$: 246.1289; found: 246.1292

2-Methyl-5-((4-Tolylamino)methyl)furan (2i)

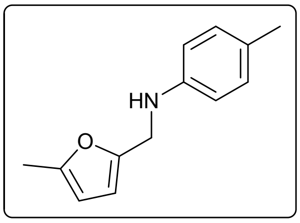

Yellow oil. Purified by flash column chromatography (cyclohexane/EtOAc = 20:1). Yield: 41% (12.4 mg).

^{1}H NMR ($CDCl_3$, 300 MHz): δ 7.03 (d, *J* = 8.2 Hz, 2H), 6.65 (d, *J* = 8.2 Hz, 2H), 6.13 (d, *J* = 2.9 Hz, 1H), 5.91 (dd, *J* = 2.9 and 0.8 Hz, 1H), 4.26 (s, 2H), 2.30 (d, *J* = 0.8 Hz, 3H), 2.27 (s, 3H).

^{13}C NMR ($CDCl_3$, 75 MHz): δ 151.60 (C), 150.74 (C), 145.20 (C), 129.69 (CH), 127.43 (C), 113.61 (CH), 107.92 (CH), 106.14 (CH), 42.05 (CH_2), 20.44 (CH_3), 13.58 (CH_3).

HRMS (ESI): calculated for $C_{13}H_{14}NO$ $(M + H)^+$: 200.1070; found: 200.1070.

1-Methyl-2-((4-Tolylamino)methyl)-1*H*-indole (2j)

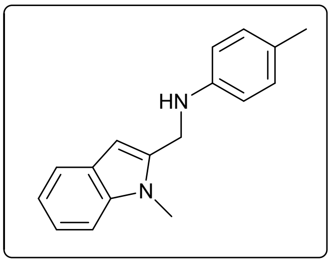

Yellow oil. Purified by flash column chromatography (cyclohexane/EtOAc = 15:1). Yield: 50% (19.0 mg).

^{1}H NMR ($CDCl_3$, 300 MHz): δ 7.59 (dt, *J* = 7.9 and 0.8 Hz, 1H), 7.34 (dd, *J* = 7.9 Hz and 0.8 Hz, 1H), 7.24 (qd, *J* = 7.0 and 1.2 Hz, 1H), 7.12 (qd, *J* = 7.0 and 1.2 Hz, 1H), 7.06 (2H), 6.68 (2H), 6.50 (d, *J* = 0.8 Hz, 1H), 4.43 (s, 2H), 3.77 (s, 3H), 2.29 (s, 3H).

^{13}C NMR ($CDCl_3$, 75 MHz): δ 145.28 (C), 137.87 (C), 137.15 (C), 129.85 (CH), 127.66 (C), 127.39 (C), 121.59 (CH), 120.47 (CH), 119.55 (CH), 113.44 (CH), 109.07 (CH), 101.33 (CH), 41.53 (CH_2), 29.81 (CH_3), 20.46 (CH_3).

HRMS (ESI): calculated for $C_{17}H_{19}N_2$ $(M + H)^+$: 251.1542; found: 251.1540.

3-((4-Tolylamino)methyl)thiophene (2k)

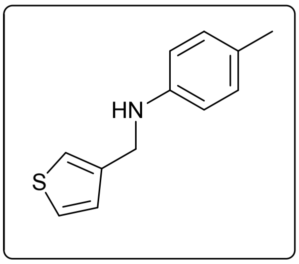

Yellow oil. Purified by flash column chromatography (cyclohexane/EtOAc = 20:1). Yield: 54% (16.3 mg).

^{1}H NMR ($CDCl_3$, 300 MHz): δ 7.32 (dd, *J* = 5.0 and 2.9 Hz, 1H), 7.20 (dd, *J* = 2.9 and 1.2 Hz, 1H), 7.10 (dd, *J* = 5.0 and 1.2 Hz, 1H), 7.03 (d, *J* = 8.1 Hz, 2H), 6.63 (d, *J* = 8.1 Hz, 2H), 4.33 (s, 2H), 2.26 (s, 3H).

^{13}C NMR ($CDCl_3$, 75 MHz): δ 145.56 (C), 140.54 (C), 129.78 (CH), 127.23 (CH), 127.15 (C), 126.09 (CH), 121.75 (CH), 113.29 (CH), 44.23 (CH_2), 20.44 (CH_3).

HRMS (ESI): calculated for $C_{12}H_{14}NS$ $(M + H)^+$: 204.0841; found: 204.0840.

3-((4-Tolylamino)methyl)pyridine (2l)

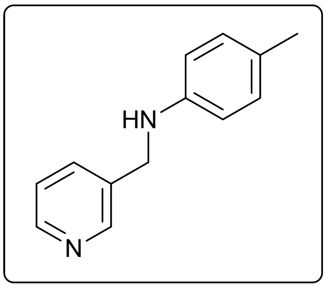

Yellow solid (m.p. = 82-83°C). Purified by flash column chromatography (cyclohexane/EtOAc = 1:1 to 1:2). Yield: 57% (17.1 mg).

^{1}H NMR ($CDCl_3$, 300 MHz): δ 8.64 (s, 1H), 8.53 (d, *J* = 4.6 Hz, 1H), 7.71 (d, *J* = 7.7 Hz, 1H), 7.28 (dd, *J* = 7.7 and 4.6 Hz, 1H), 7.00 (d, *J* = 8.3 Hz, 2H), 6.56 (d, *J* = 8.3 Hz, 2H), 4.35 (s, 2H), 3.56 (bs, 1H), 2.25 (s, 3H).

^{13}C NMR ($CDCl_3$, 75 MHz): δ 149.01 (CH), 148.49 (CH), 145.31 (C), 135.27 (CH and C), 129.83 (CH), 127.31 (C), 123.60 (CH), 113.12 (CH), 46.11 (CH_2), 20.40 (CH_3).

HRMS (ESI): calculated for $C_{13}H_{15}N_2$ $(M + H)^+$: 199.1230; found: 199.1227.

***N*-(4-Isopropylbenzyl)aniline (2m)**

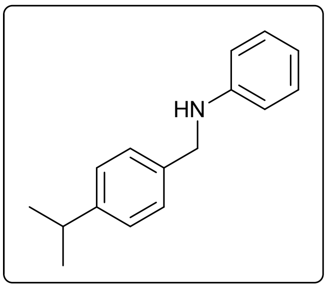

Yellow oil. Purified by flash column chromatography (cyclohexane/EtOAc = 20:1). Yield: 57% (19.1 mg).

^{1}H NMR ($CDCl_3$, 300 MHz): δ 7.33 (d, *J* = 8.1 Hz, 2H), 7.26-7.17 (m, 4H), 6.75 (t, *J* = 7.3 Hz, 1H), 6.68 (d, *J* = 8.1 Hz, 2H), 4.31 (s, 2H), 2.94 (hep, *J* = 6.9 Hz, 1H), 1.28 (d, *J* = 6.9 Hz, 6H).

^{13}C NMR ($CDCl_3$, 75 MHz): δ 148.09 (C), 148.02 (C), 136.61 (C), 129.28 (CH), 127.70 (CH), 126.70 (CH), 117.64 (CH), 112.96 (CH), 48.22 (CH_2), 33.84 (CH), 24.07 (CH_3).

HRMS (ESI): calculated for $C_{16}H_{20}N$ $(M + H)^+$: 226.1590; found: 226.1591.

***N*-((4-Isopropylbenzyl)-naphthylamine (2n)**

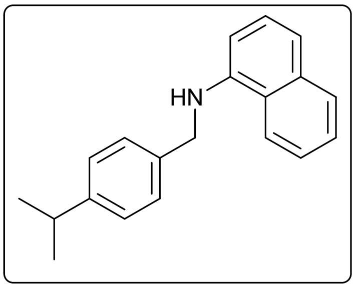

Brown oil. Purified by flash column chromatography (cyclohexane/EtOAc = 20:1). Yield: 56% (23.1 mg).

^{1}H NMR ($CDCl_3$, 300 MHz): δ 7.85 (t, *J* = 7.6 Hz, 2H), 7.53-7.36 (m, 5H), 7.33-7.25 (m, 3H), 6.71 (d, *J* = 7.5 Hz, 1H), 4.48 (s, 2H), 2.96 (hep, *J* = 6.9 Hz, 1H), 1.30 (d, *J* = 6.9 Hz, 6H).

^{13}C NMR ($CDCl_3$, 75 MHz): δ 148.22 (C), 134.29 (C), 128.69 (CH), 127.98 (CH), 126.80 (CH), 126.62 (CH), 125.76 (CH), 124.77 (CH), 123.43 (C), 119.99 (CH), 48.57 (CH_2), 33.87 (CH), 24.07 (CH_3).

HRMS (ESI): calculated for $C_{20}H_{22}N$ $(M + H)^+$: 276.1747; found: 276.1747.

_N_-((4-Isopropylbenzyl)-3-methoxyaniline (2o)

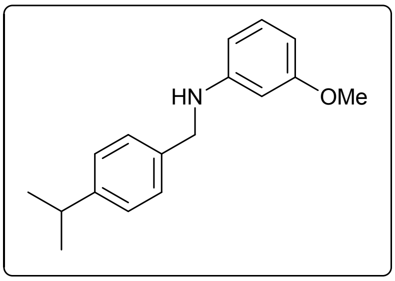

Yellow oil. Purified by flash column chromatography (cyclohexane/EtOAc = 20:1). Yield: 64% (24.6 mg).

^{1}H NMR ($CDCl_3$, 300 MHz): δ 7.33 (d, *J* = 8.1 Hz, 2H), 7.23 (d, *J* = 8.1 Hz, 2H), 7.11 (t, *J* = 8.0 Hz, 1H), 6.35-6.27 (m, 2H), 6.24 (t, *J* = 2.2 Hz, 1H), 4.30 (s, 2H), 3.78 (s, 3H), 2.94 (hep, *J* = 6.9 Hz, 1H), 1.29 (d, *J* = 6.9 Hz, 6H).

^{13}C NMR ($CDCl_3$, 75 MHz): δ 160.83 (C), 149.59 (C), 148.02 (C), 136.58 (C), 130.00 (CH), 127.70 (CH), 126.71 (CH), 106.04 (CH), 102.67 (CH), 98.87 (CH), 55.09 (CH_3), 48.17 (CH_2), 33.84 (CH), 24.07 (CH_3).

HRMS (ESI): calculated for $C_{17}H_{22}NO$ $(M + H)^+$: 256.1696; found: 256.1697.

Methyl 3-((4-isopropylbenzyl)amino)benzoate (2p)

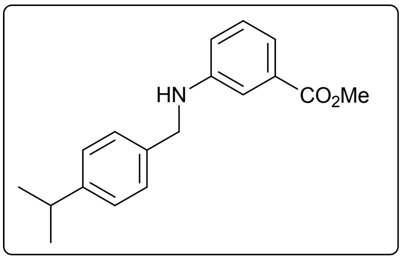

Yellowish solid (m.p. = 72-74 °C). Purified by flash column chromatography (cyclohexane/EtOAc = 30:1). Yield: 58% (24.4 mg).

^{1}H NMR ($CDCl_3$, 300 MHz): δ 7.44-7.19 (m, 7H), 6.82 (ddd, *J* = 0.9, 2.5 and 8.1 Hz, 1H), 4.33 (s, 2H), 3.90 (s, 3H), 2.93 (hep, *J* = 6.9 Hz, 1H), 1.28 (d, *J* = 6.9 Hz, 6H).

^{13}C NMR ($CDCl_3$, 75 MHz): δ 167.48 (C), 148.19 (C), 148.01 (C), 136.07 (C), 131.06 (C), 129.21 (CH), 127.72 (CH), 126.76 (CH), 118.77 (CH), 117.36 (CH), 113.59 (CH), 52.04 (CH_3), 48.13 (CH_2), 33.82 (CH), 24.03 (CH_3).

HRMS (ESI): calculated for $C_{18}H_{22}NO_2$ $(M + H)^+$: 284.1645; found: 284.1648.

4. Mechanistic studies

Experiment 1: Into an undivided beaker-type screening electrolysis cell equipped with a glassy carbon anode and a Pb cathode, CH_3CN (2.5 mL), water (2.5 mL), Na_2CO_3 (42.4 mg, 0.4 mmol), NBu_4BF_4 (32.9 mg, 0.01 mmol), and freshly prepared nitrone **1a** (37.9 mg, 0.15 mmol) were added. A constant current electrolysis with a current density of 30 mA/cm^2 (I = 50 mA) was performed at room temperature. After application of 44C (3F) the electrolysis was stopped. The mixture was then transferred into a separation funnel, whereby AcOEt (20 mL) and water (20 mL) were added. The aqueous layer was again extracted with AcOEt (20 mL), the combined organic fractions were washed with brine (20 mL) and dried over $MgSO_4$. The solution was concentrated under reduced pressure and the ^{1}H NMR of the crude was recorded (Figure 3). We could observed the remaining nitrone (singlet marked in red at 7.89 ppm), the imine (singlet marked in green at 8.44 ppm) and the amine (singlet marked in blue at 4.28 ppm), in a proportion 47 / 7 / 46 (The value of the singlet of the amine was divided by 2).

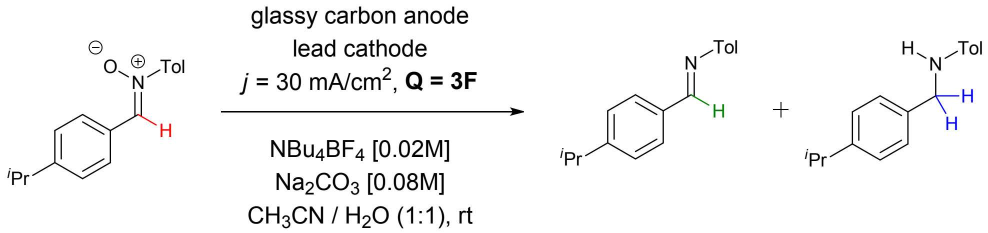

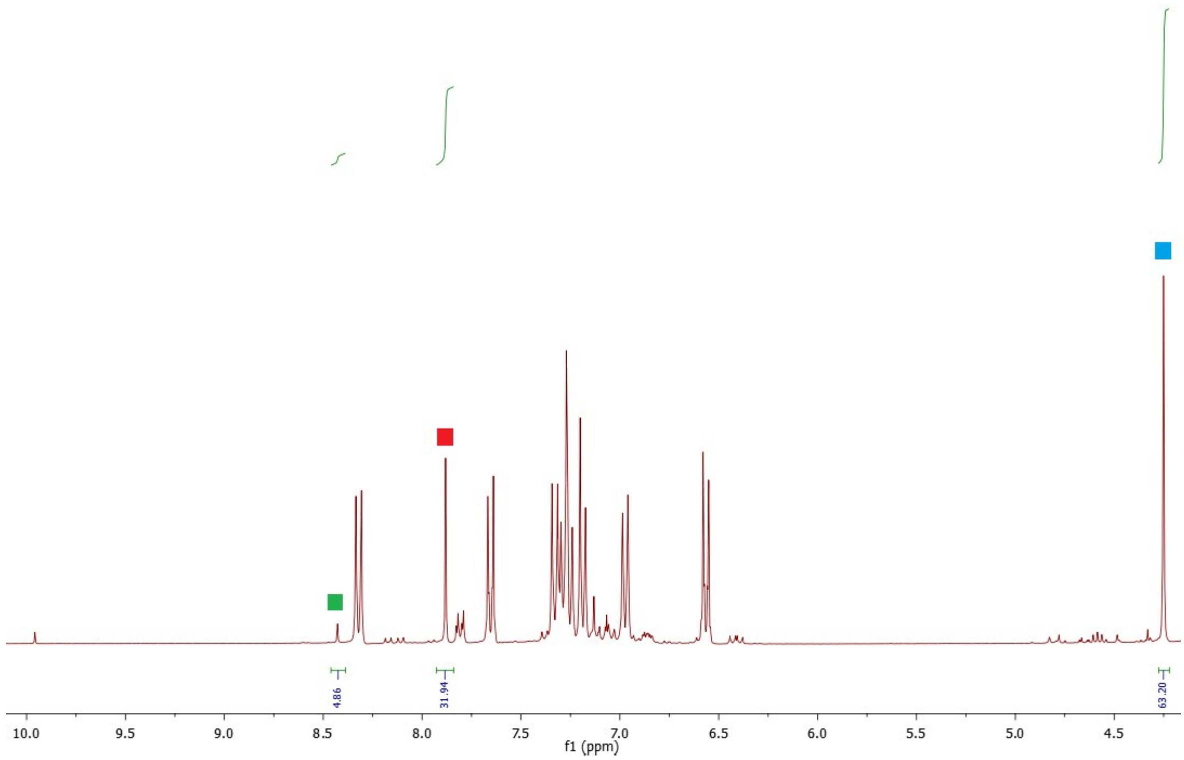

Figure 3. ^{1}H NMR of the electrolysis at Q = 3F.

Experiment 2: Into an undivided beaker-type screening electrolysis cell equipped with a glassy carbon anode and a Pb cathode, CH_3CN (12.5 mL), water (12.5 mL), Na_2CO_3 (210 mg, 2 mmol), NBu_4BF_4 (165 mg, 0.05 mmol), and *N*-Benzylideneaniline (136 mg, 0.75 mmol) were added. A constant current electrolysis with a current density of 30 mA/cm^2 (I = 210 mA) was performed at room temperature. An aliquot of the reaction mixture (1 mL) was taken every time at 0F (0 C), 1F (72 C), 2F (144 C) and 3F (215F) and then transferred into a separation funnel, whereby AcOEt (10 mL) and water (10 mL) were added. The aqueous layer was again extracted with AcOEt (10 mL), the combined organic fractions were washed with brine (10 mL) and dried over $MgSO_4$. The solution was concentrated under reduced pressure and the crude of every aliquot was recorded by ^{1}H NMR (Figure 4).

We could observed the evolution of the reaction. The imine (Singlet in red at 8.46 ppm) leads to the formation of the amine (singlet in blue at 4.32 ppm), while there is also formation of benzaldehyde (singlet in green at 10.03 ppm) and benzyl alcohol (singlet in orange at 4.66 ppm). This was confirmed with the m/z value (108) found in the GC-MS for one of the major peaks.

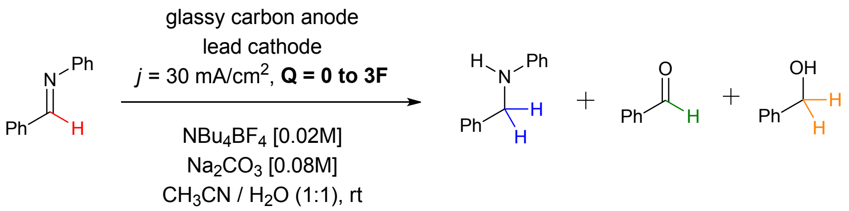

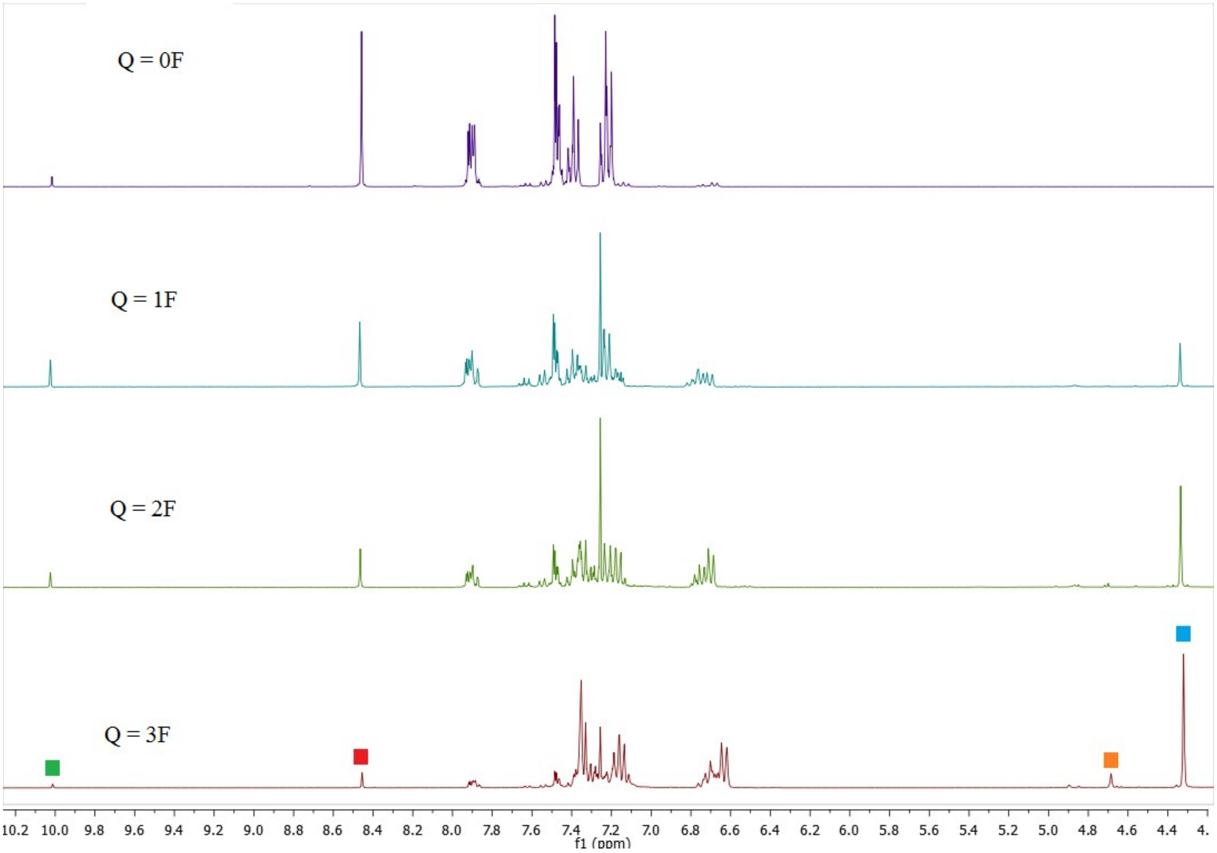

Figure 4. ^{1}H NMR of the electrolysis of *N*-Benzylideneaniline at Q = 0F – 3F.

5. ^{1}H and ^{13}C spectra of amines 2

***N*-(4-Isopropylbenzyl)-4-methylaniline (2a)**

(^{1}H NMR, $CDCl_3$, 300 MHz)

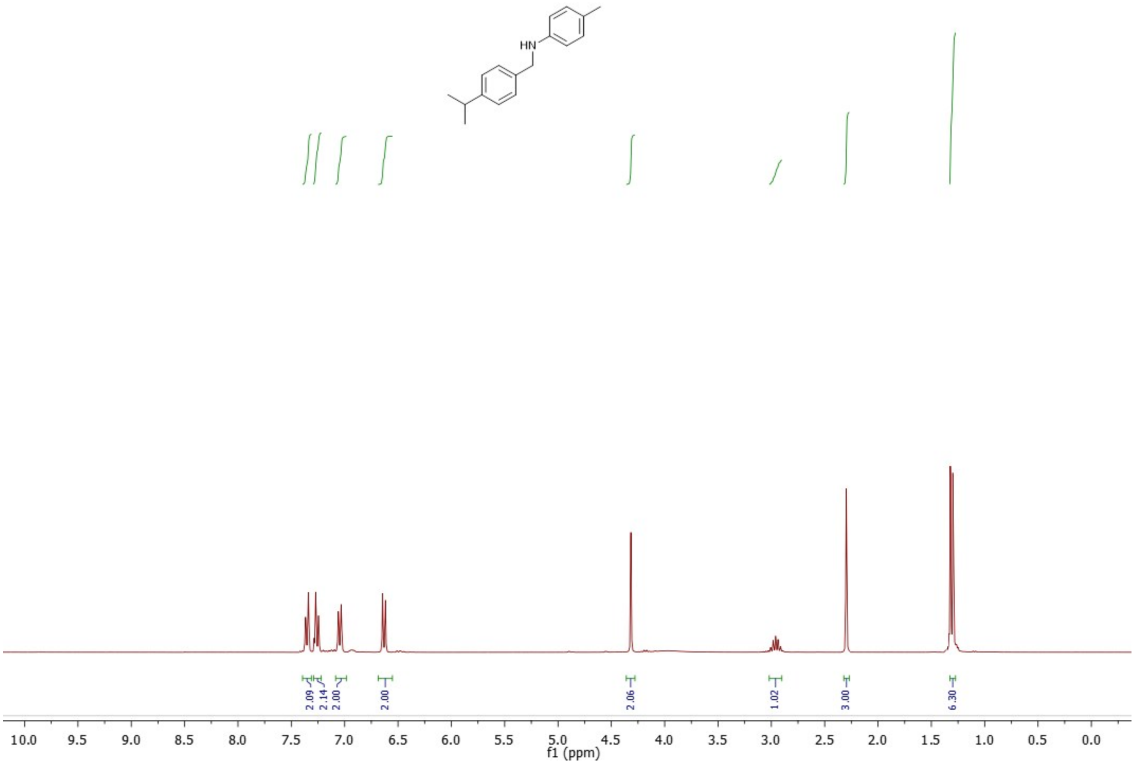

(^{13}C NMR, $CDCl_3$, 75 MHz)

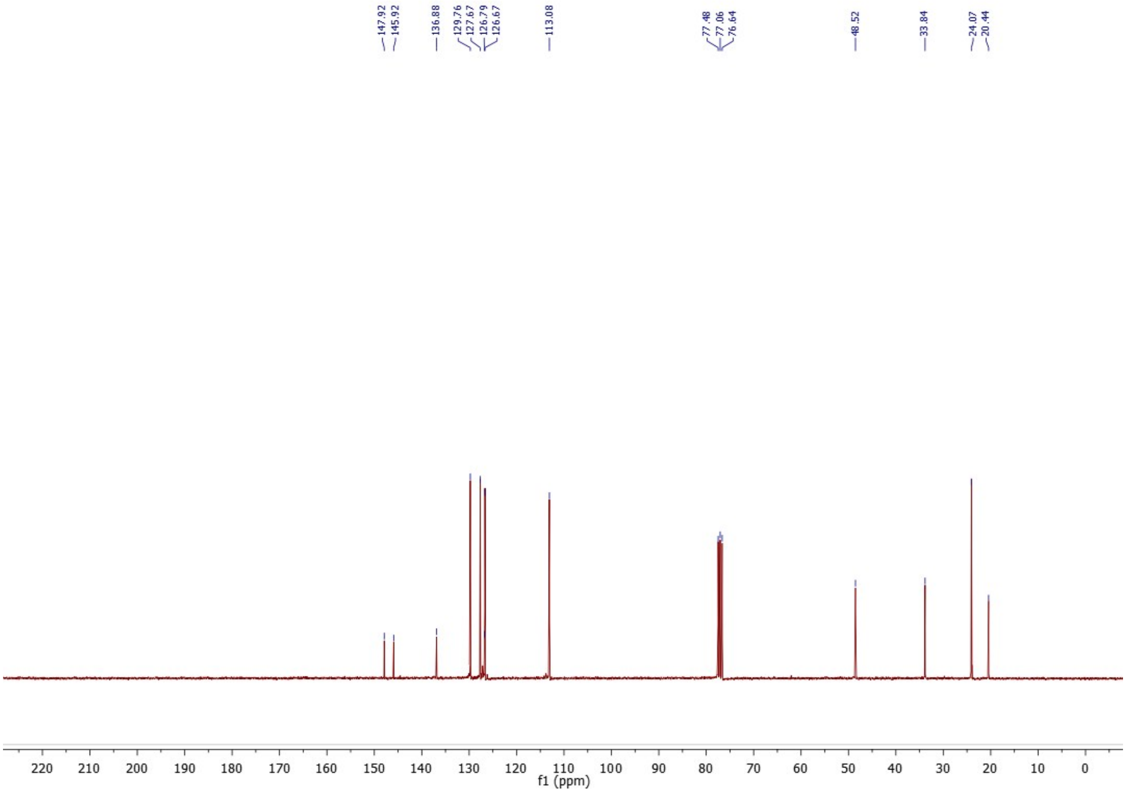

4-((4-Tolylamino)methyl)benzonitrile (2b)

(^{1}H NMR, $CDCl_3$, 300 MHz)

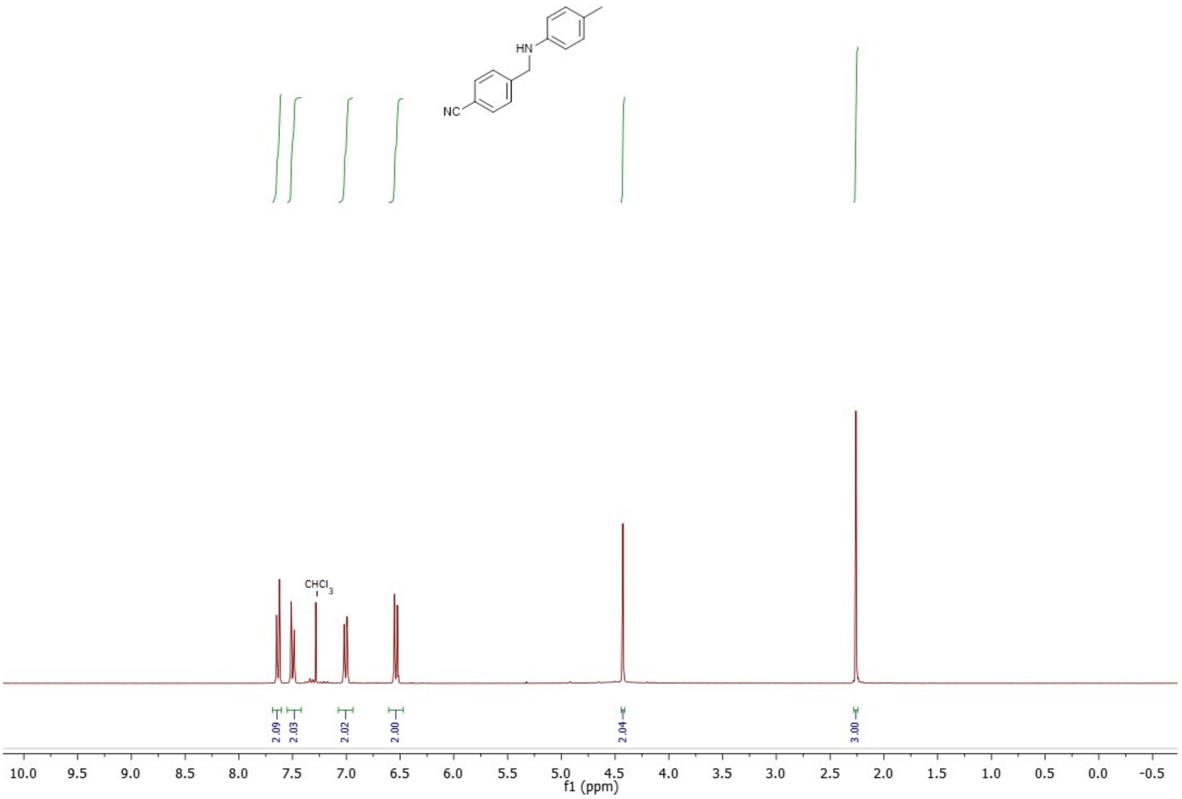

(^{13}C NMR, $CDCl_3$, 75 MHz)

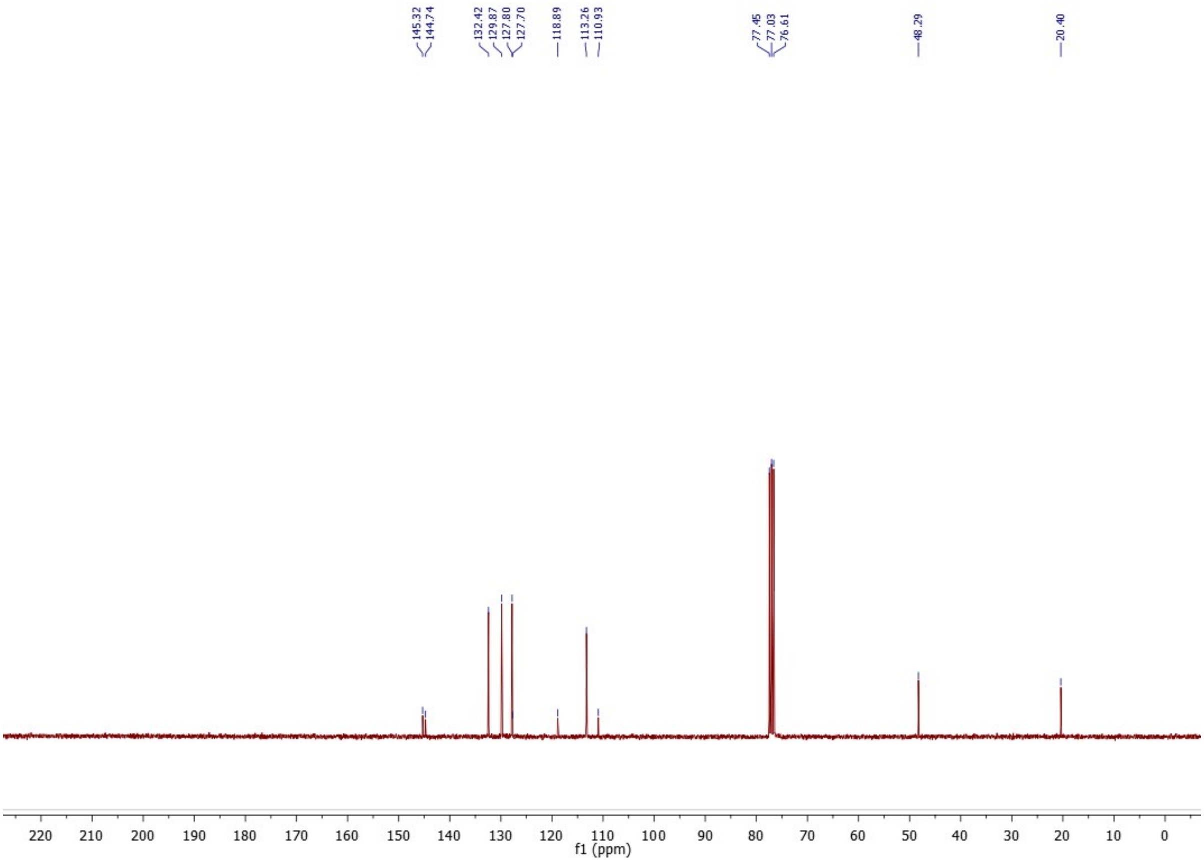

4-Methyl-*N*-(4-(methylsulfanyl)benzyl)aniline (2c)

(^{1}H NMR, $CDCl_3$, 300 MHz)

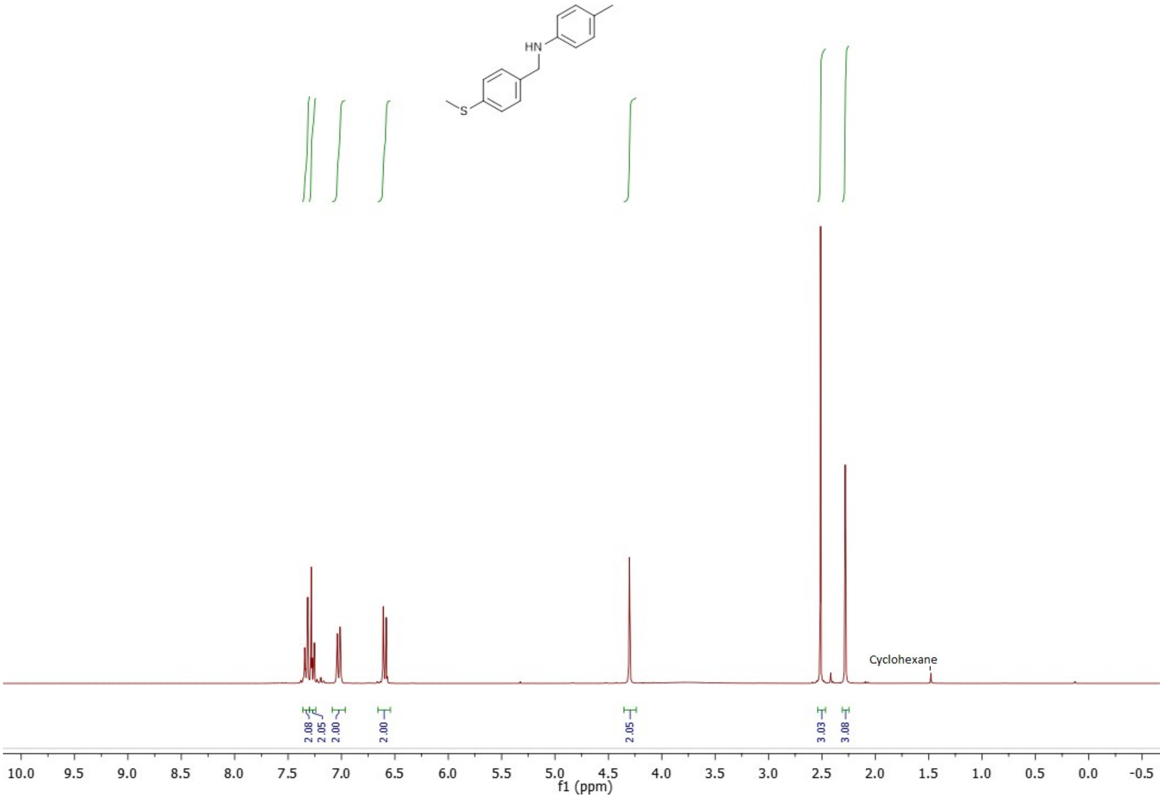

(^{13}C NMR, $CDCl_3$, 75 MHz)

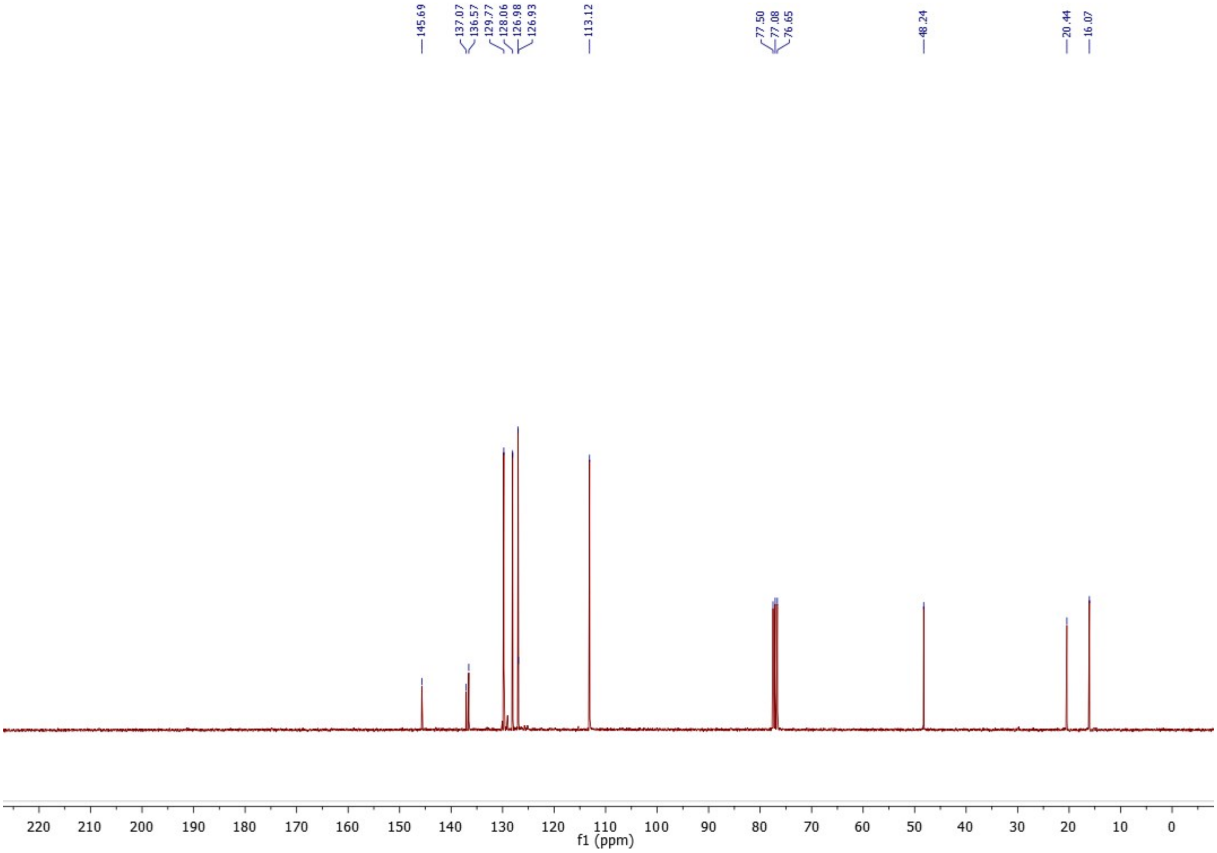

***N,N*-Dimethyl-4-((4-tolylamino)methyl)aniline (2d)**

(^{1}H NMR, $CDCl_3$, 300 MHz)

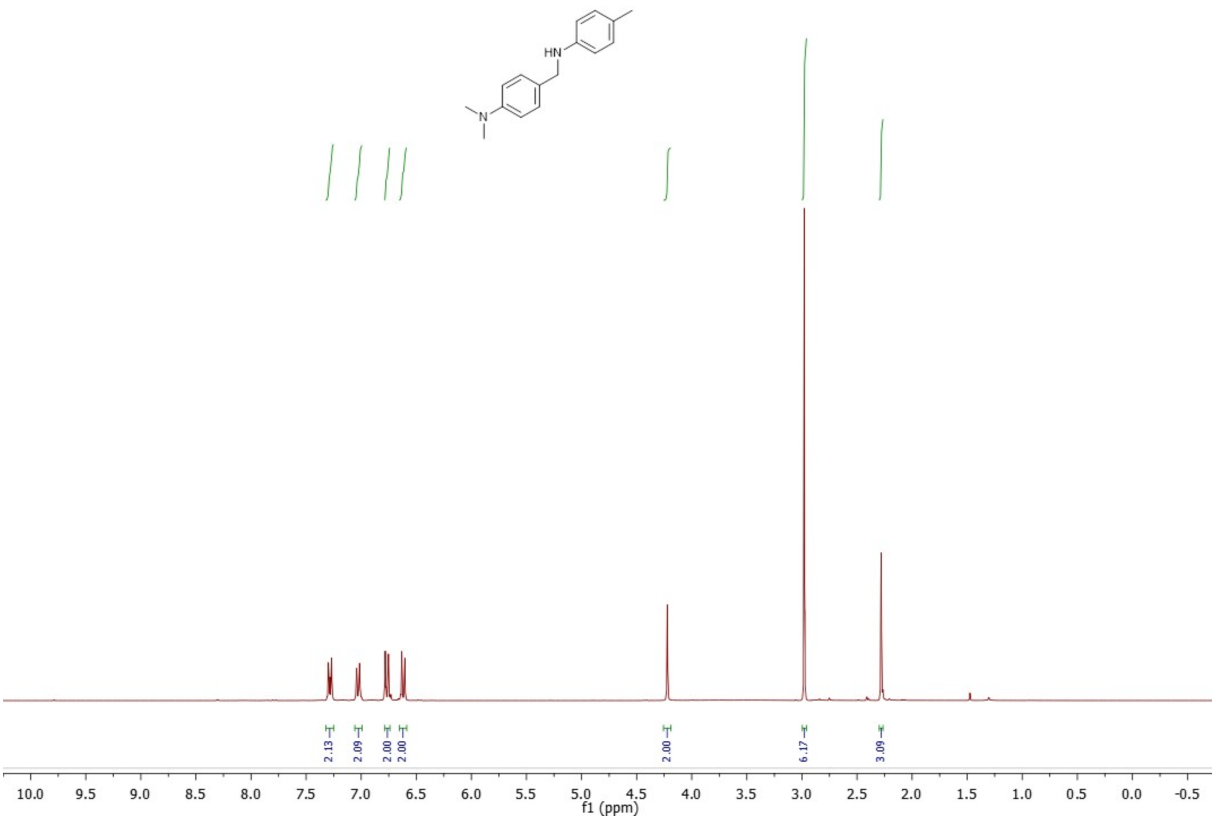

(^{13}C NMR, $CDCl_3$, 75 MHz)

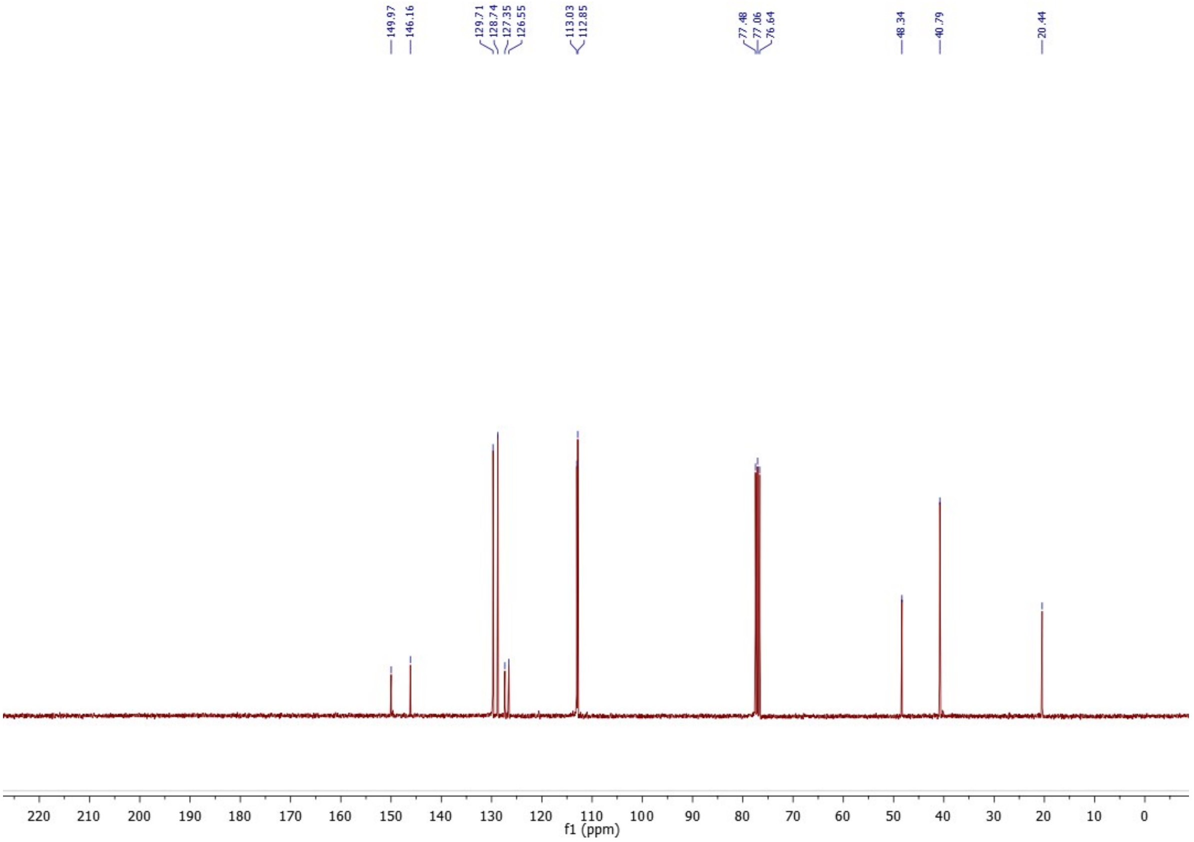

4-Methyl-*N*-(naphth-2-ylmethyl)aniline (2e)

(^1H NMR, $CDCl_3$, 300 MHz)

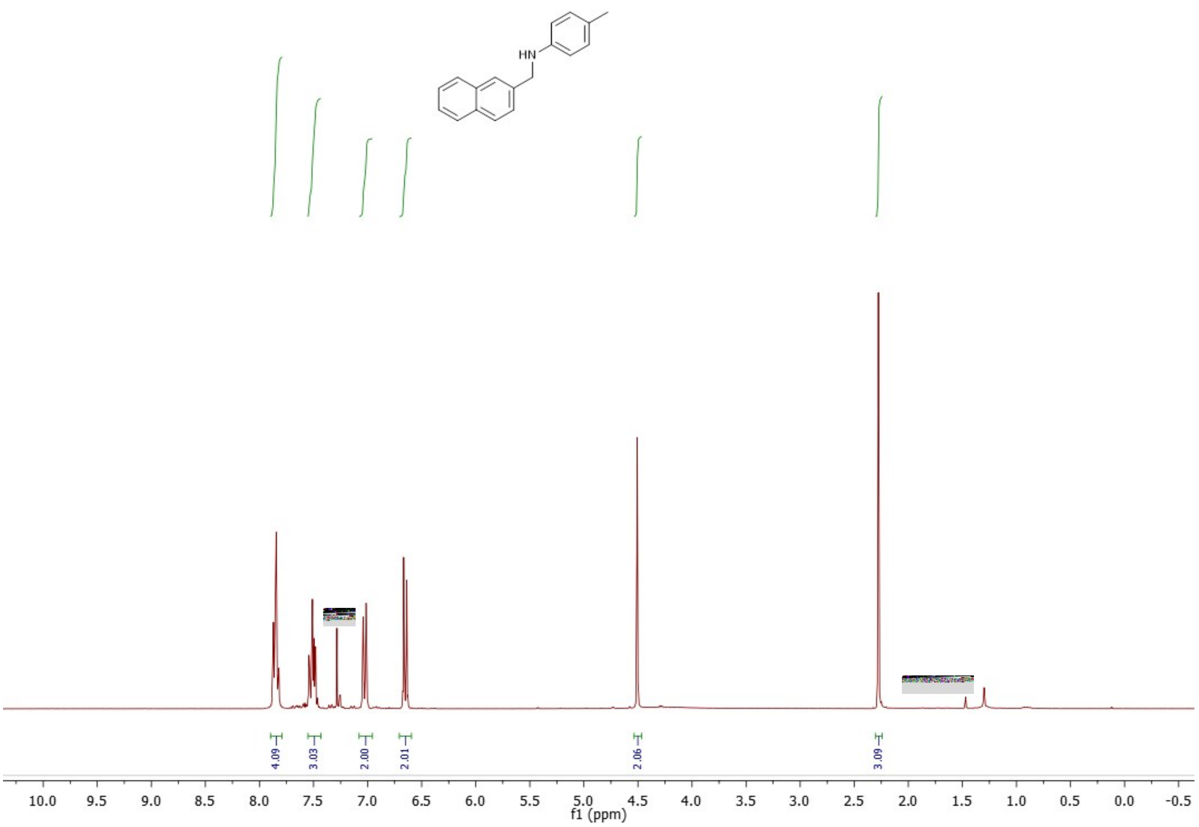

(^{13}C NMR, $CDCl_3$, 75 MHz)

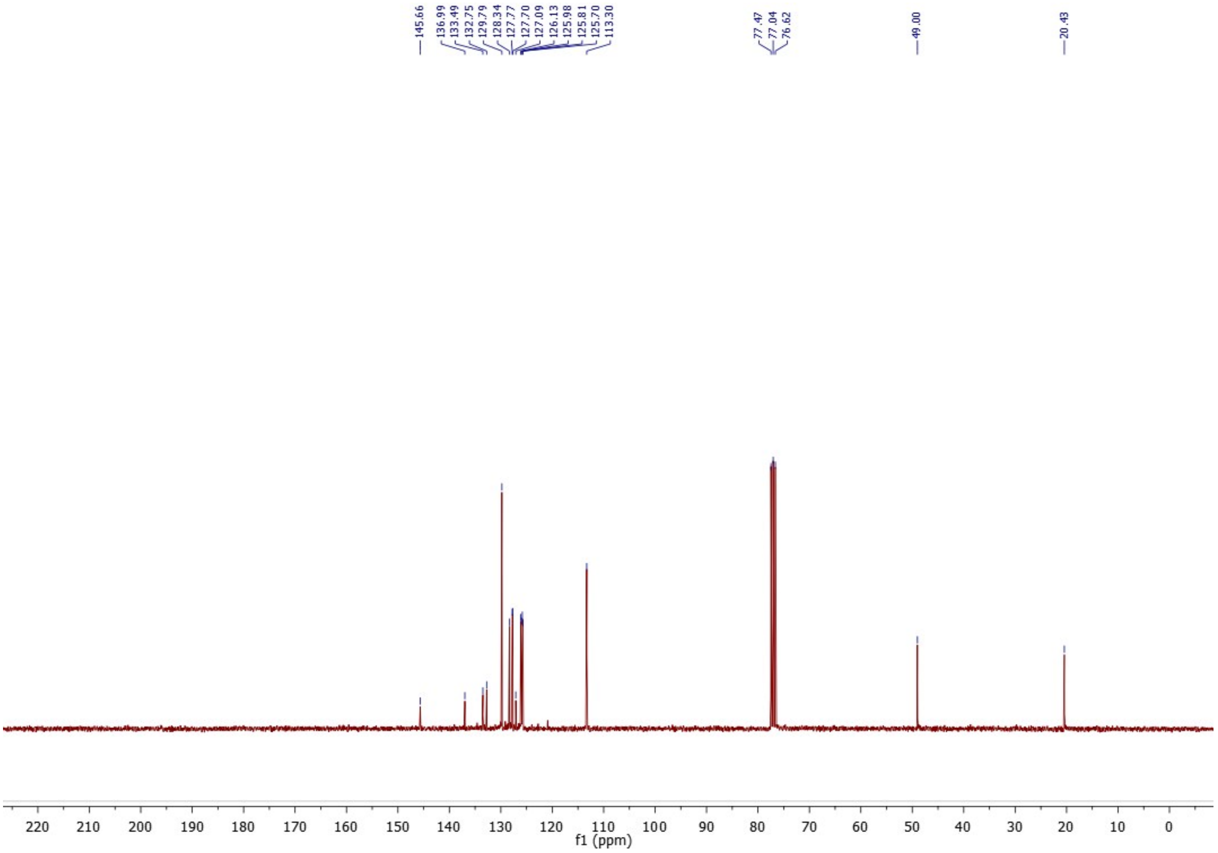

***N*-(2,3-Dimethoxybenzyl)-4-methylaniline (2f)**

(^{1}H NMR, $CDCl_3$, 300 MHz)

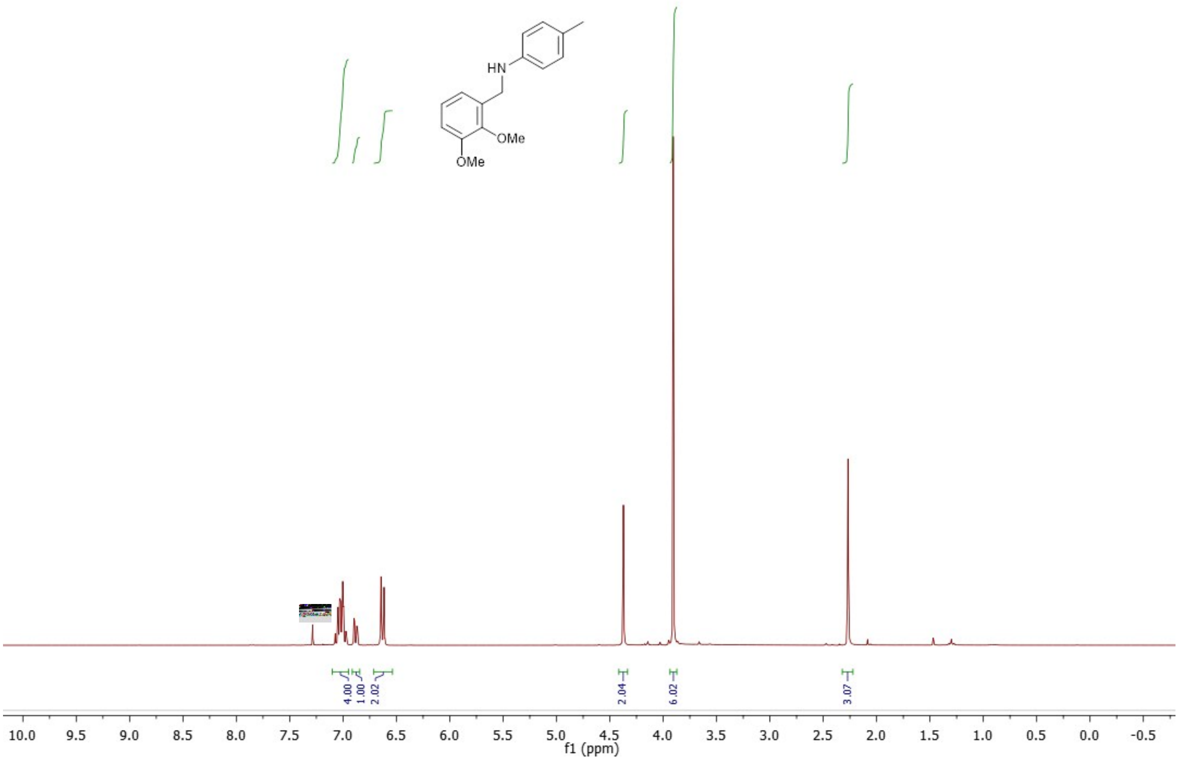

(^{13}C NMR, $CDCl_3$, 75 MHz)

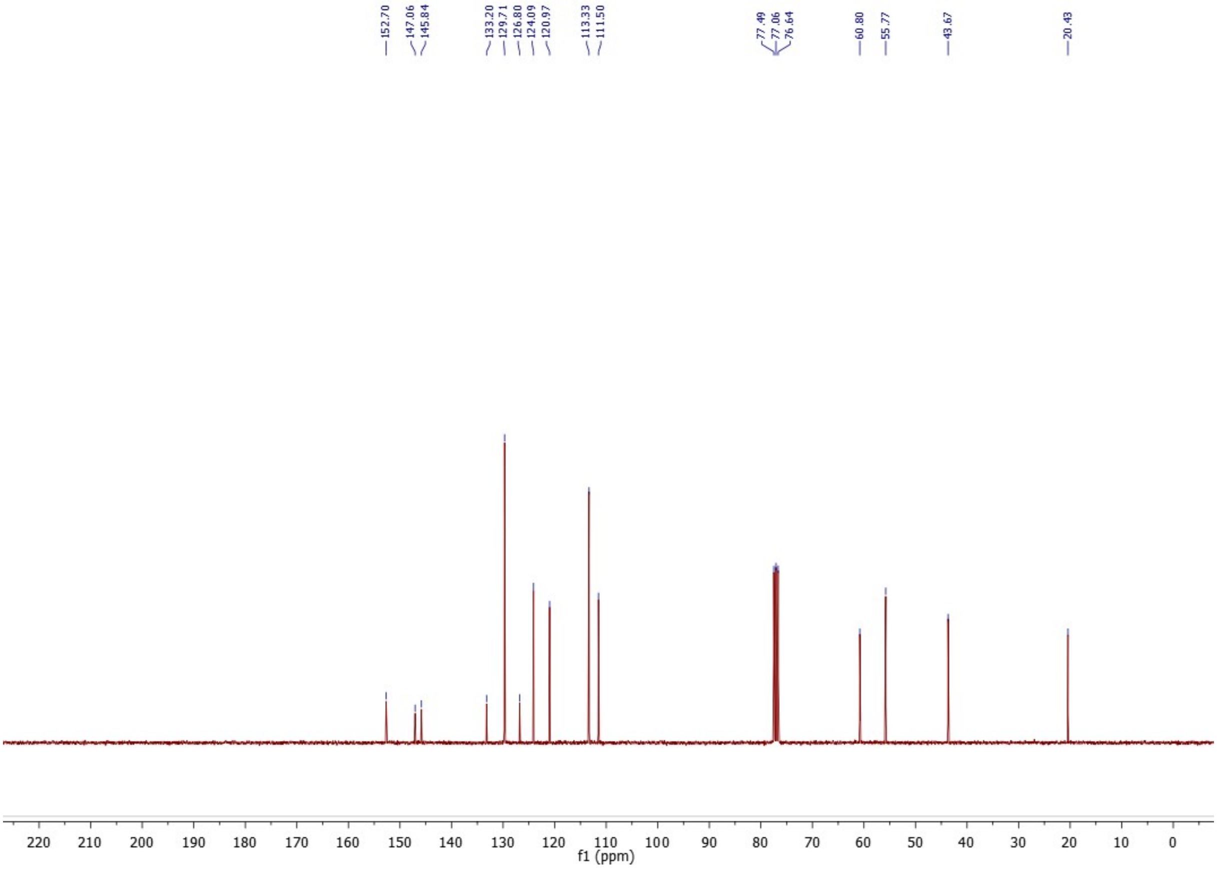

***N*-(2,6-Dimethylbenzyl)-4-methylaniline (2g)**

(^{1}H NMR, $CDCl_3$, 300 MHz)

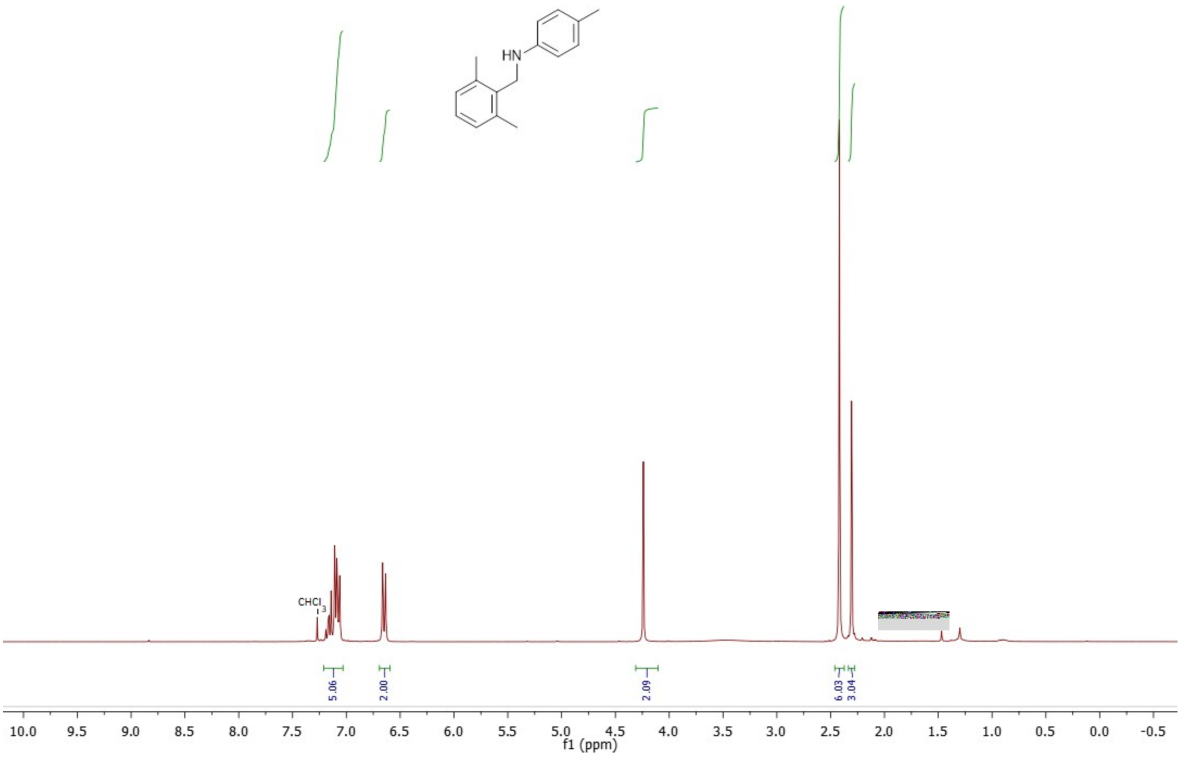

(^{13}C NMR, $CDCl_3$, 75 MHz)

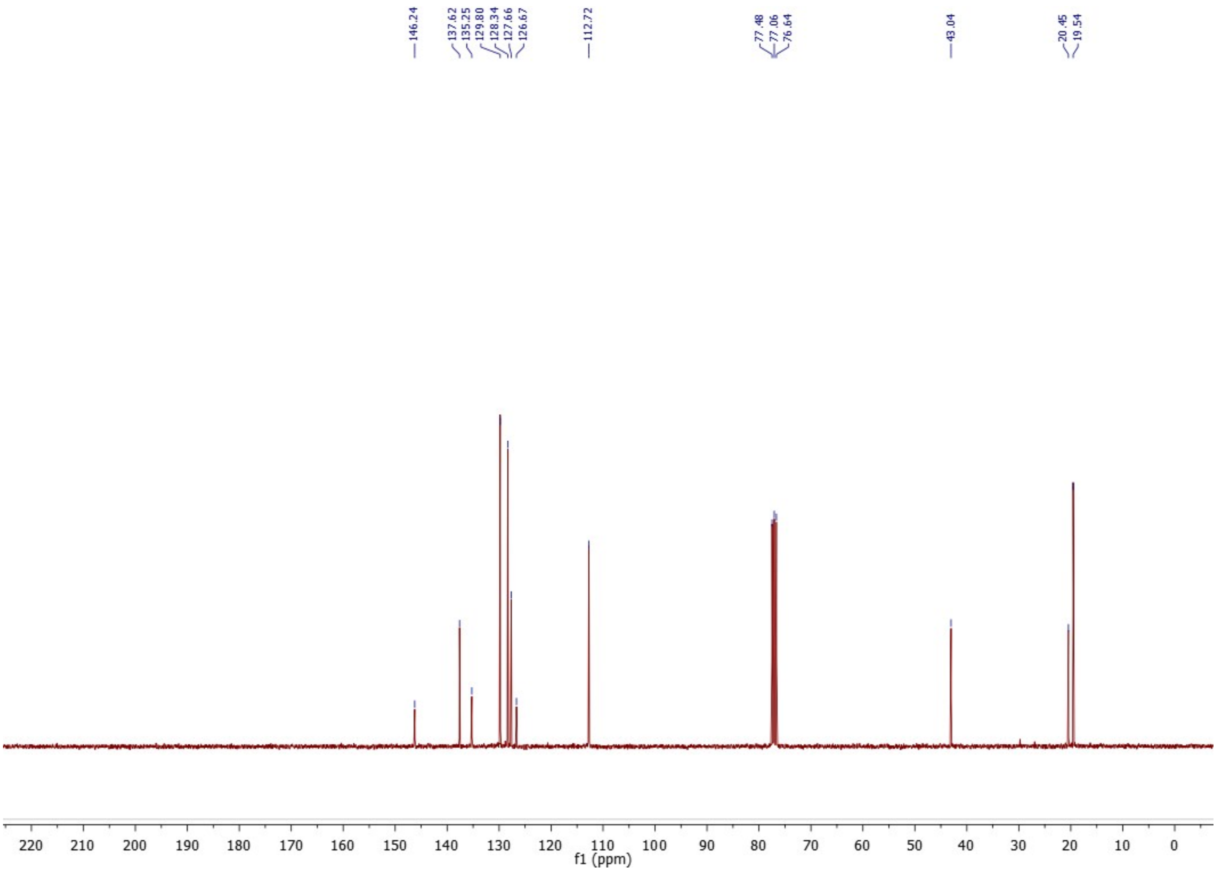

_N_-(3-Fluoro-4-methoxybenzyl)-4-methylaniline (2h)

(^{1}H NMR, $CDCl_3$, 300 MHz)

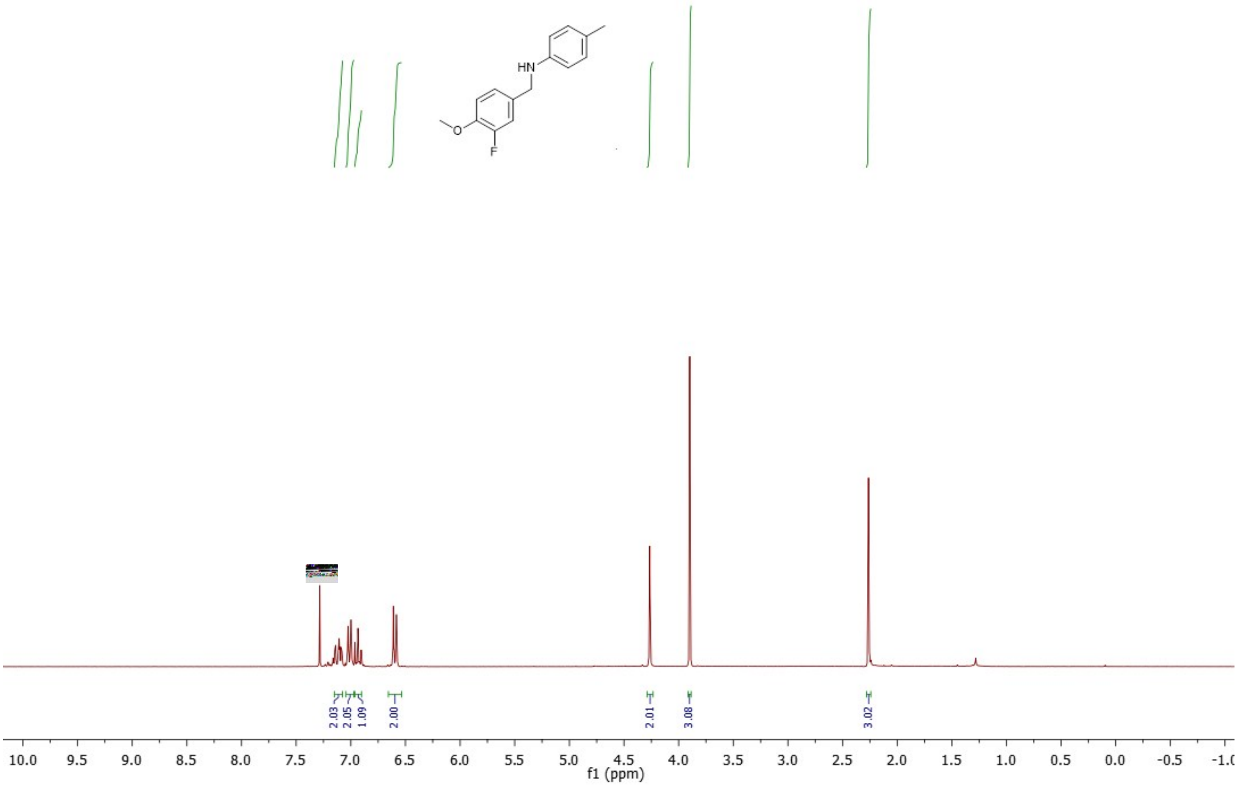

(^{13}C NMR, $CDCl_3$, 75 MHz)

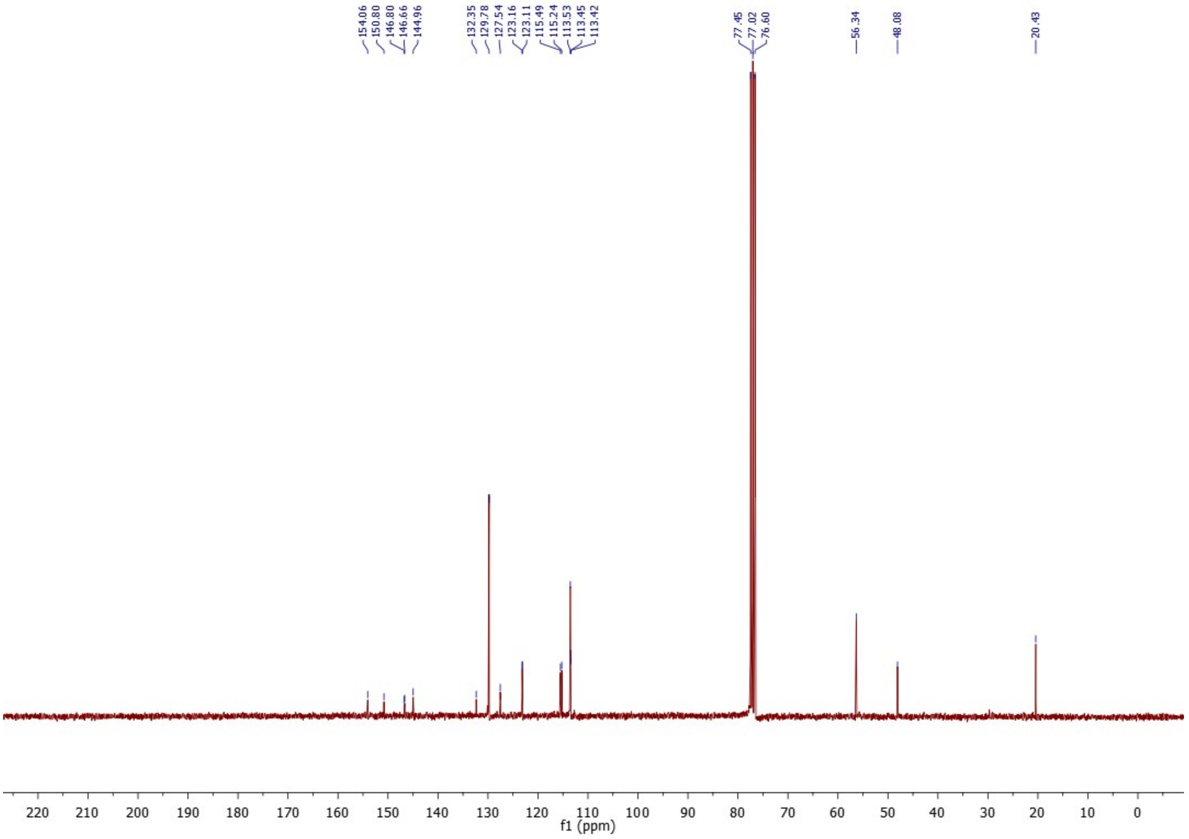

2-Methyl-5-((4-Tolylamino)methyl)furan (2i)

(^1H NMR, $CDCl_3$, 300 MHz)

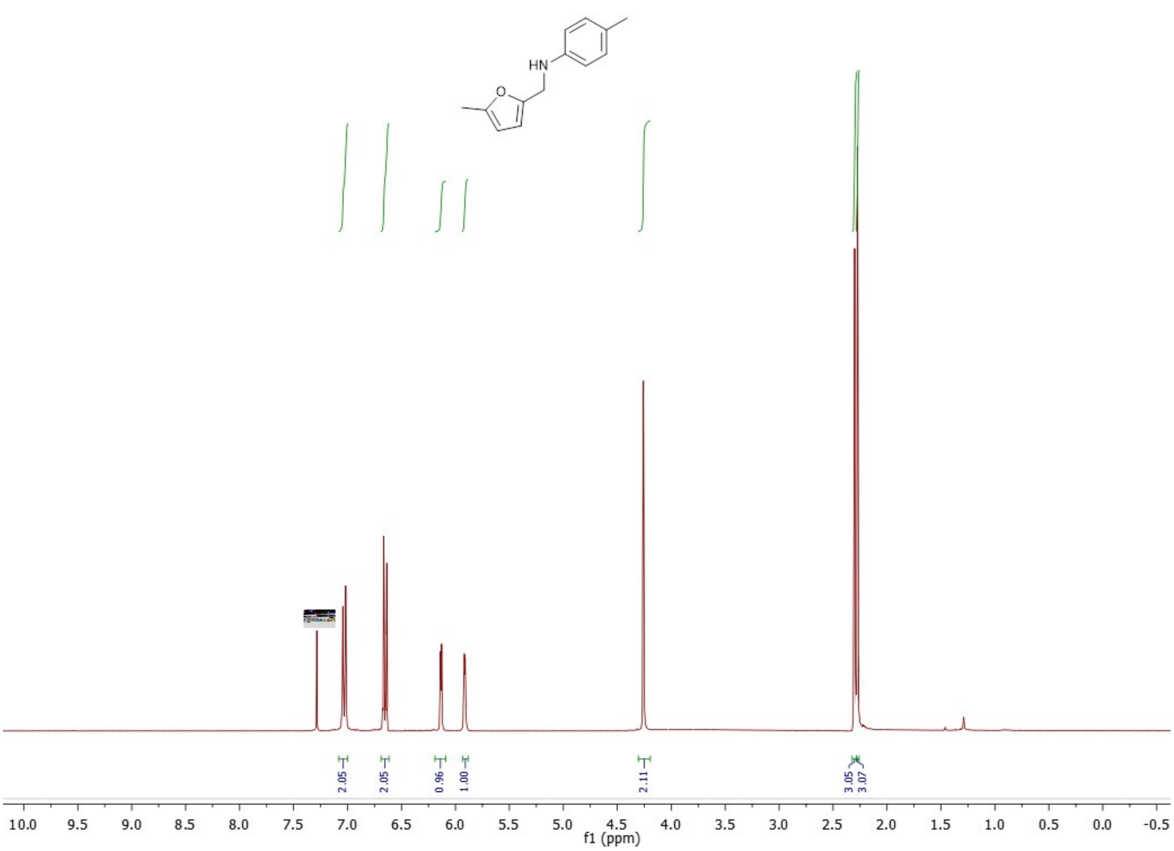

(^{13}C NMR, $CDCl_3$, 75 MHz)

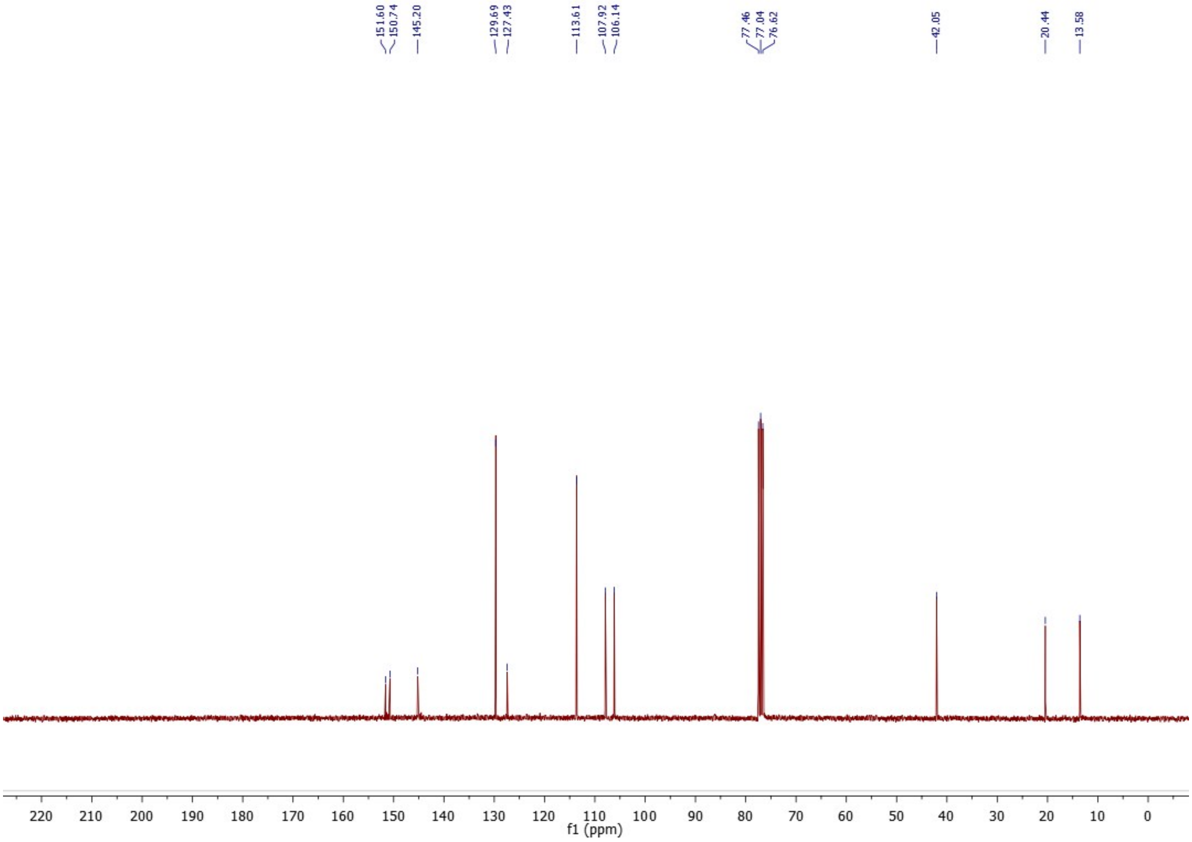

1-Methyl-2-((4-Tolylamino)methyl)-1*H*-indole (2j)

(^{1}H NMR, $CDCl_3$, 300 MHz)

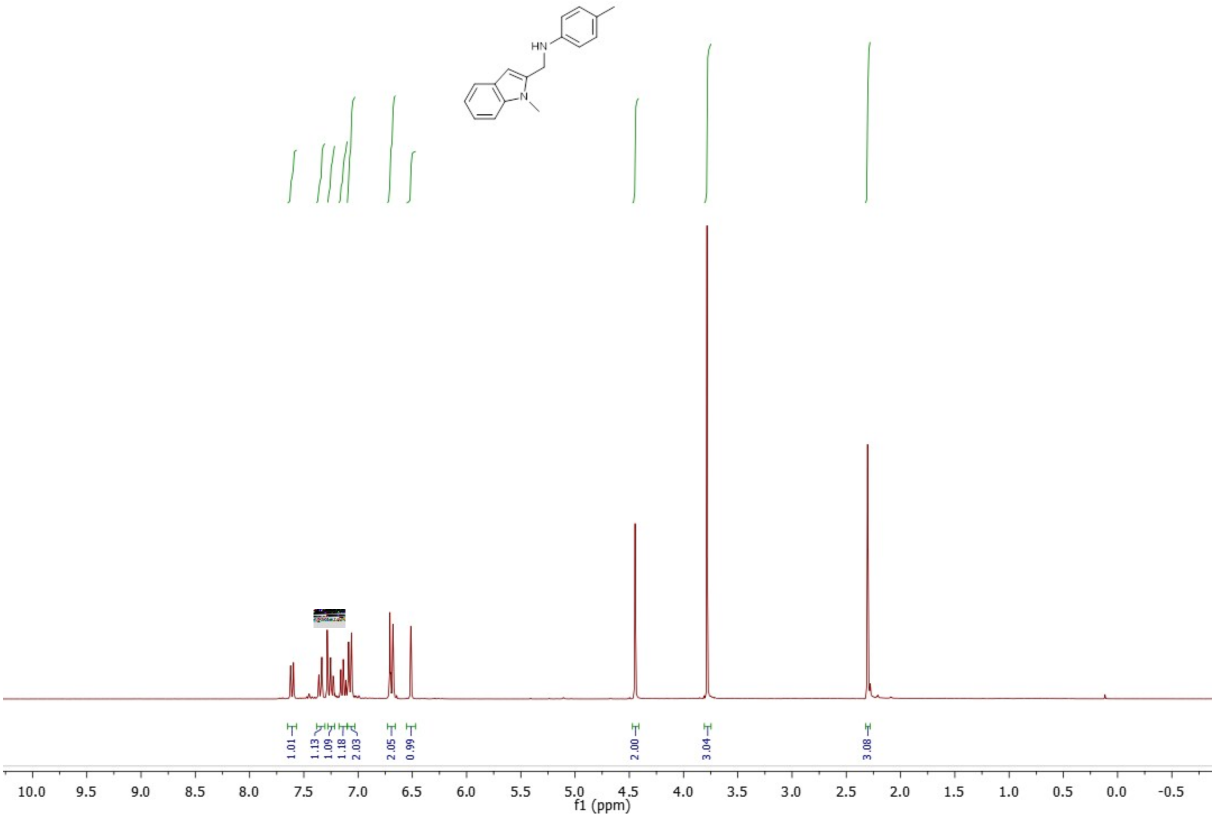

(^{13}C NMR, $CDCl_3$, 75 MHz)

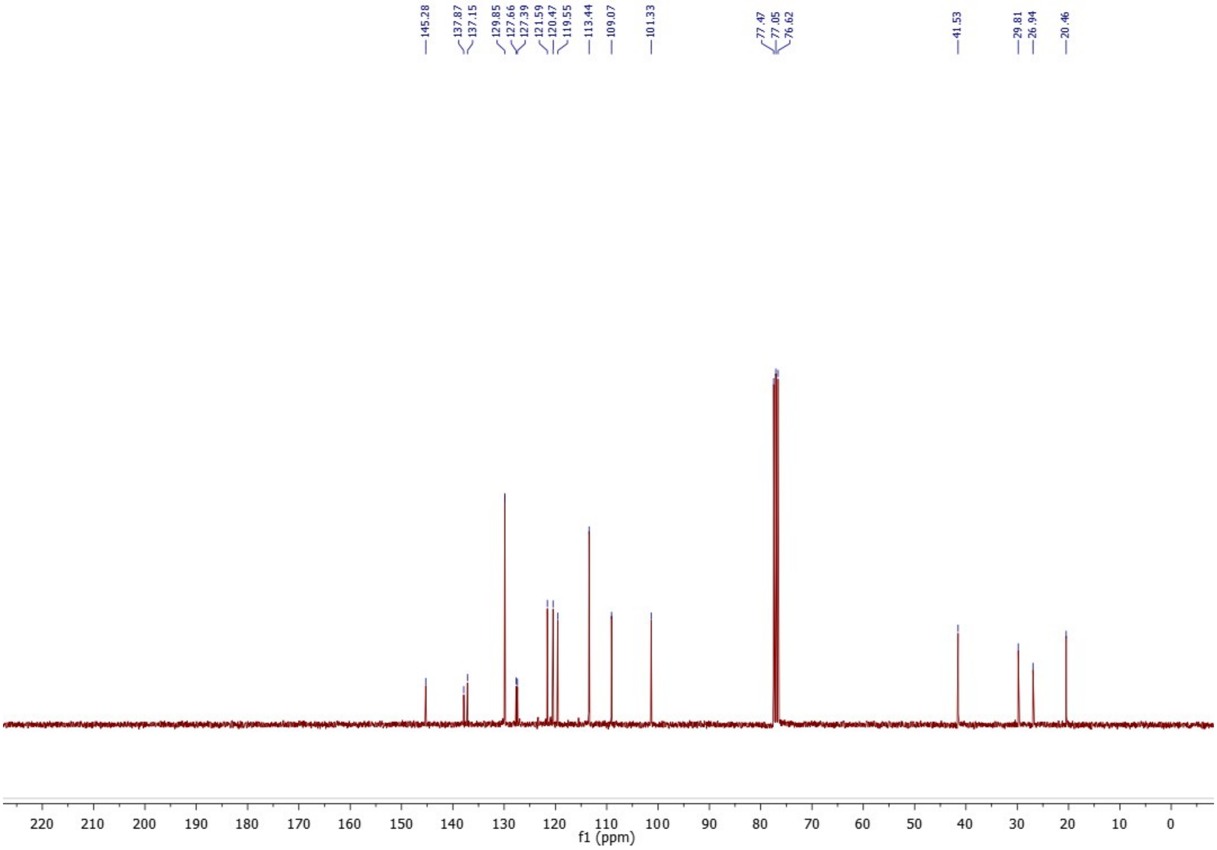

3-((4-Tolylamino)methyl)thiophene (2k)

(^{1}H NMR, $CDCl_3$, 300 MHz)

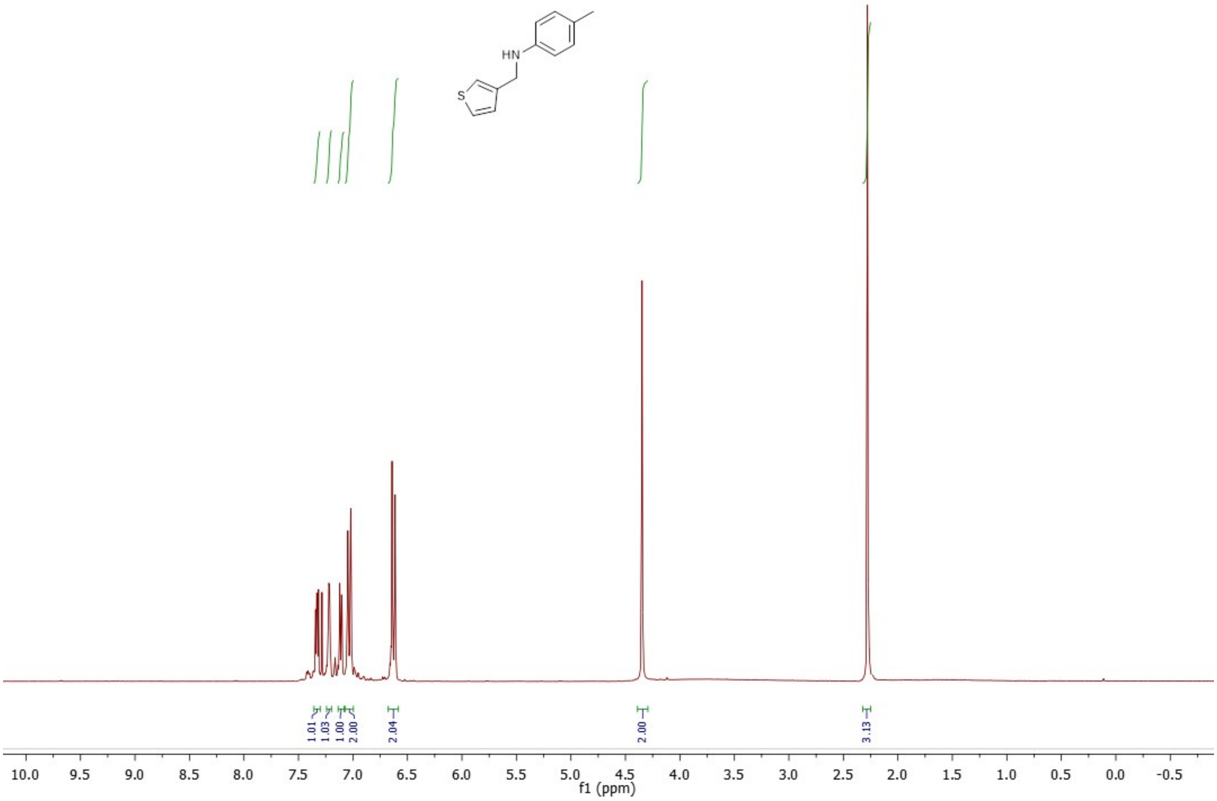

(^{13}C NMR, $CDCl_3$, 75 MHz)

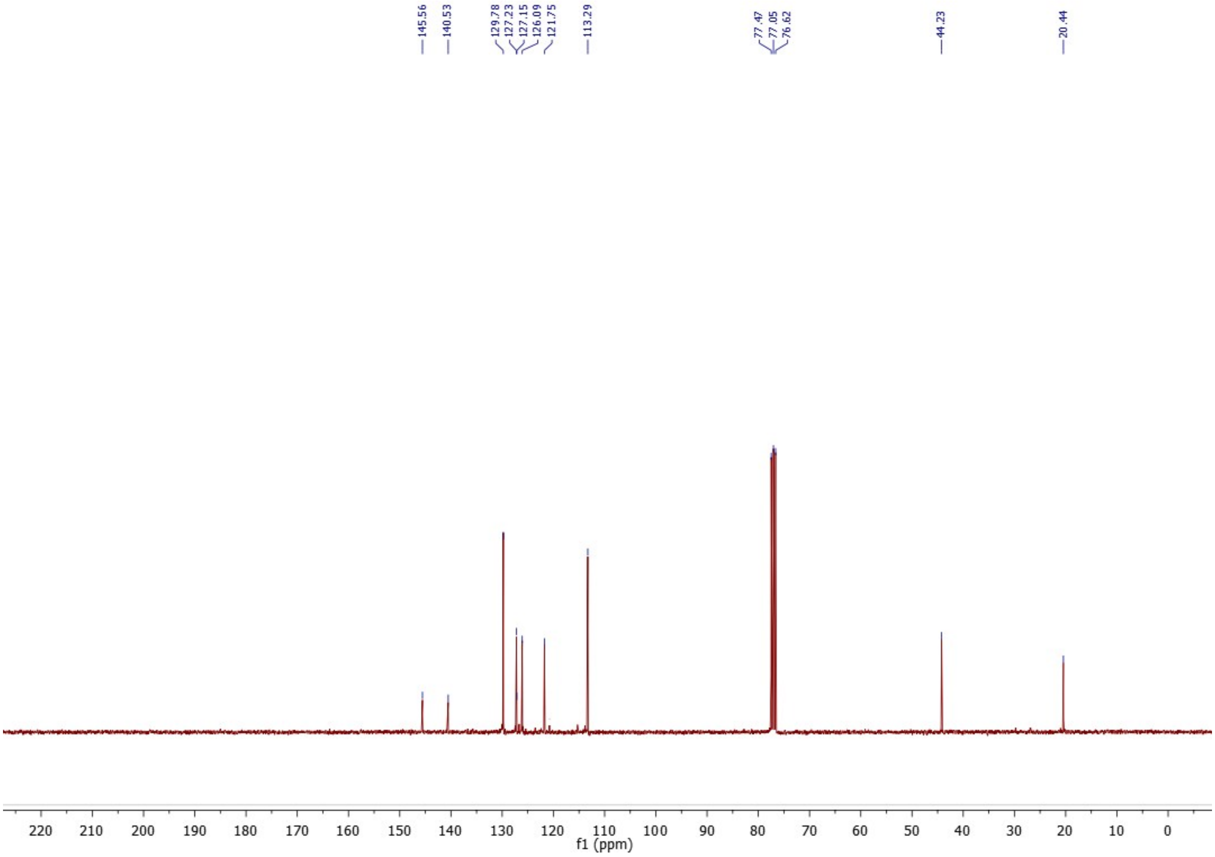

3-((4-Tolylamino)methyl)pyridine (2l)

(^{1}H NMR, $CDCl_3$, 300 MHz)

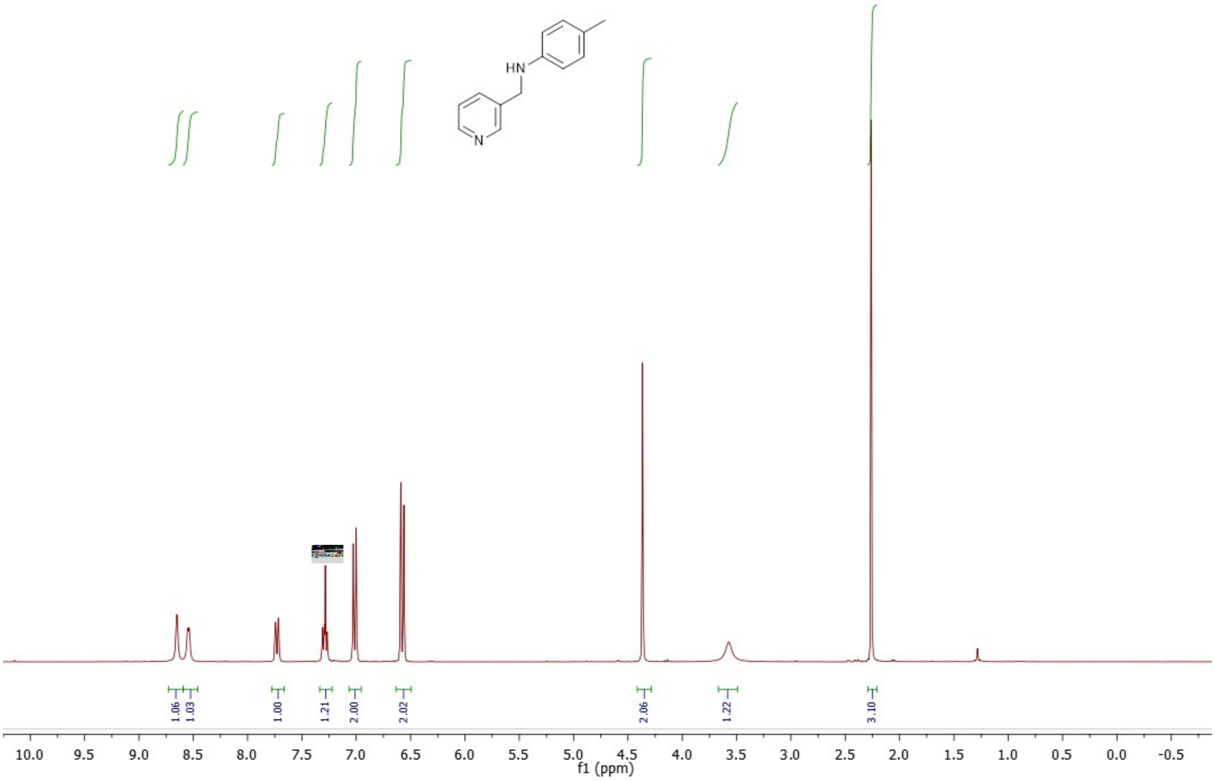

(^{13}C NMR, $CDCl_3$, 75 MHz)

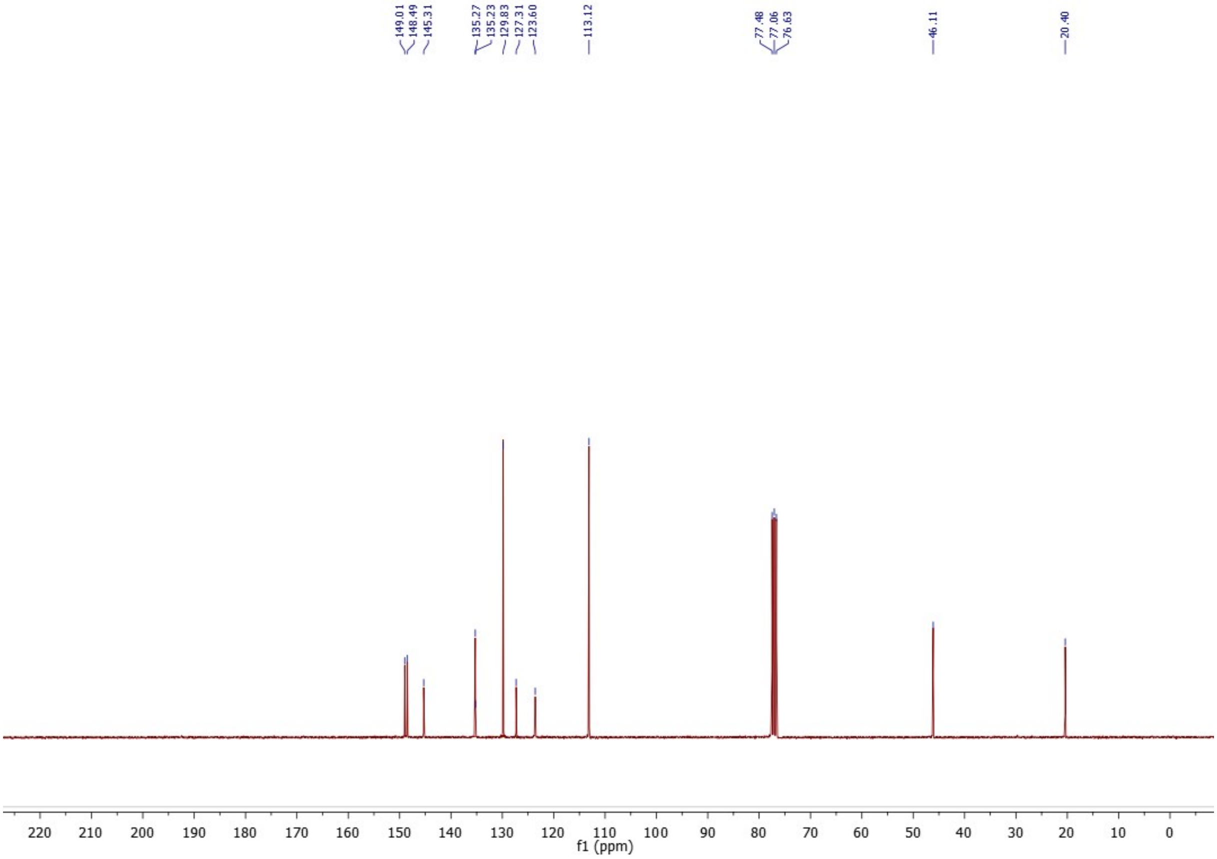

***N*-(4-Isopropylbenzyl)aniline (2m)**

(^1H NMR, $CDCl_3$, 300 MHz)

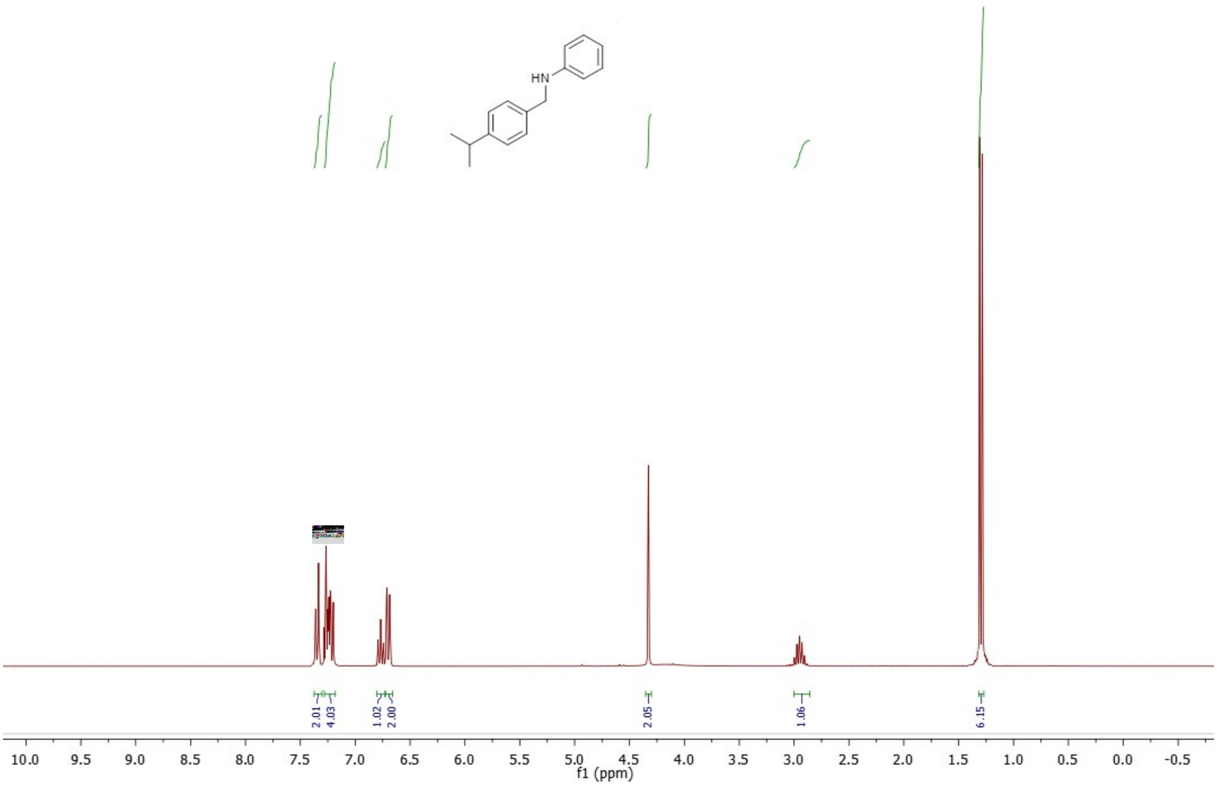

(^{13}C NMR, $CDCl_3$, 75 MHz)

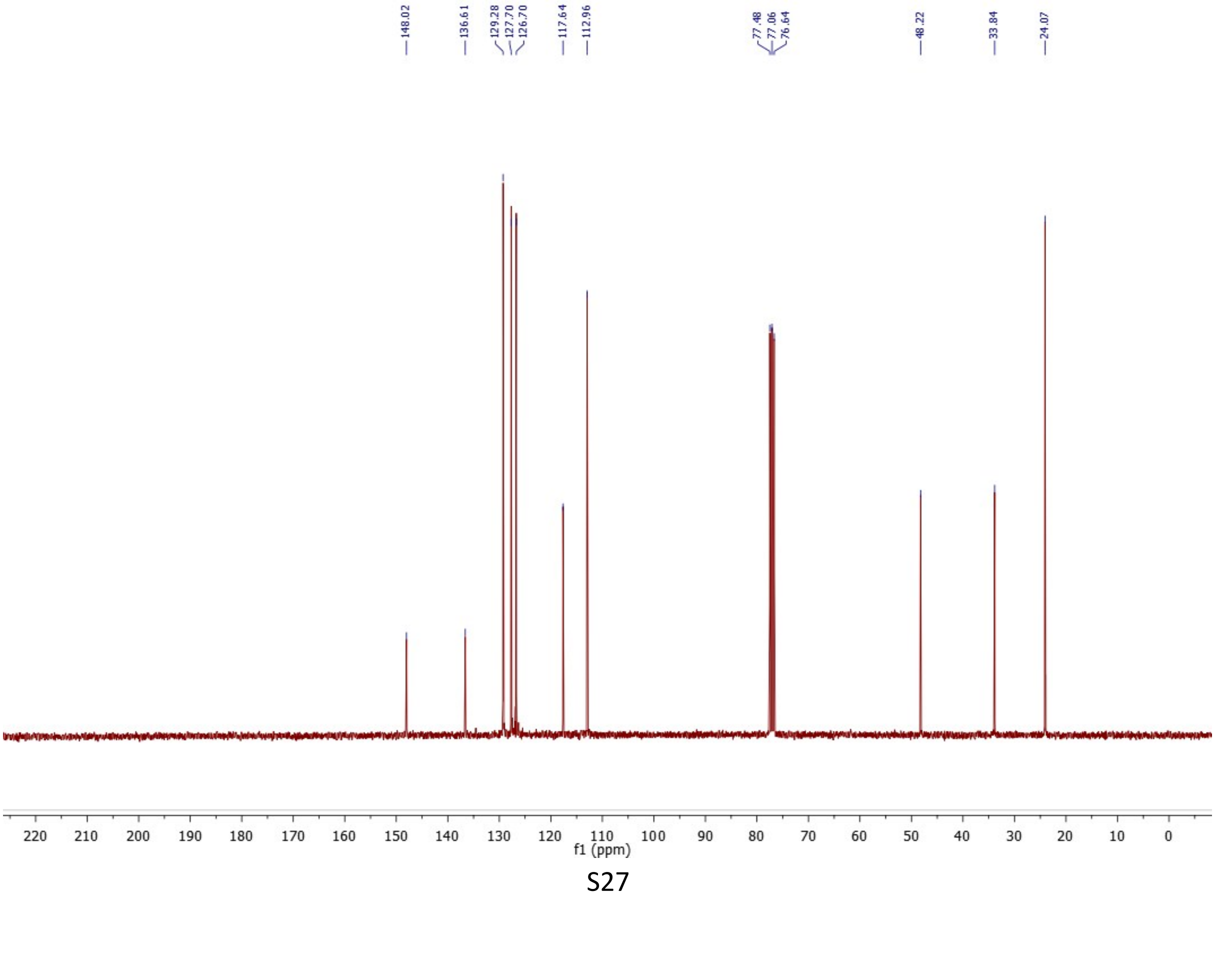

***N*-((4-Isopropylbenzyl)-naphthylamine (2n)**

(^{1}H NMR, $CDCl_3$, 300 MHz)

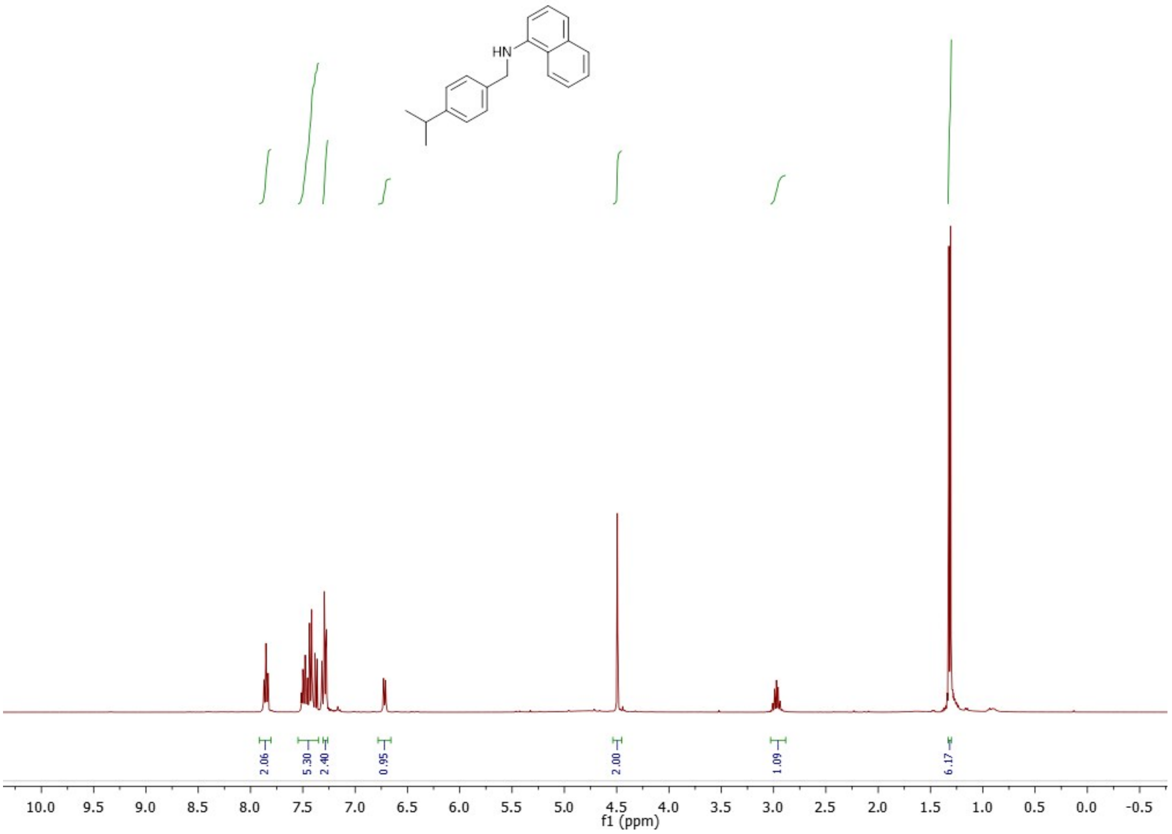

(^{13}C NMR, $CDCl_3$, 75 MHz)

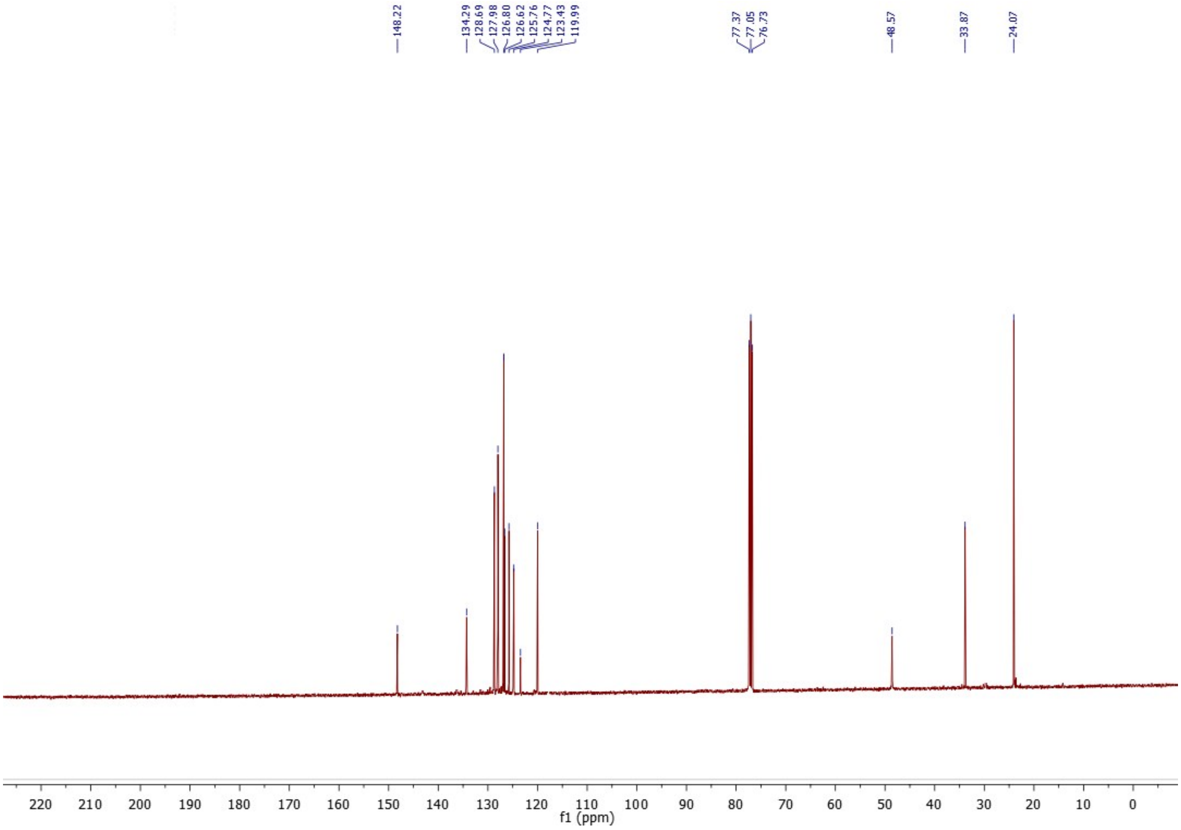

***N*-((4-Isopropylbenzyl)-3-methoxyaniline (2o)**

(^{1}H NMR, $CDCl_3$, 300 MHz)

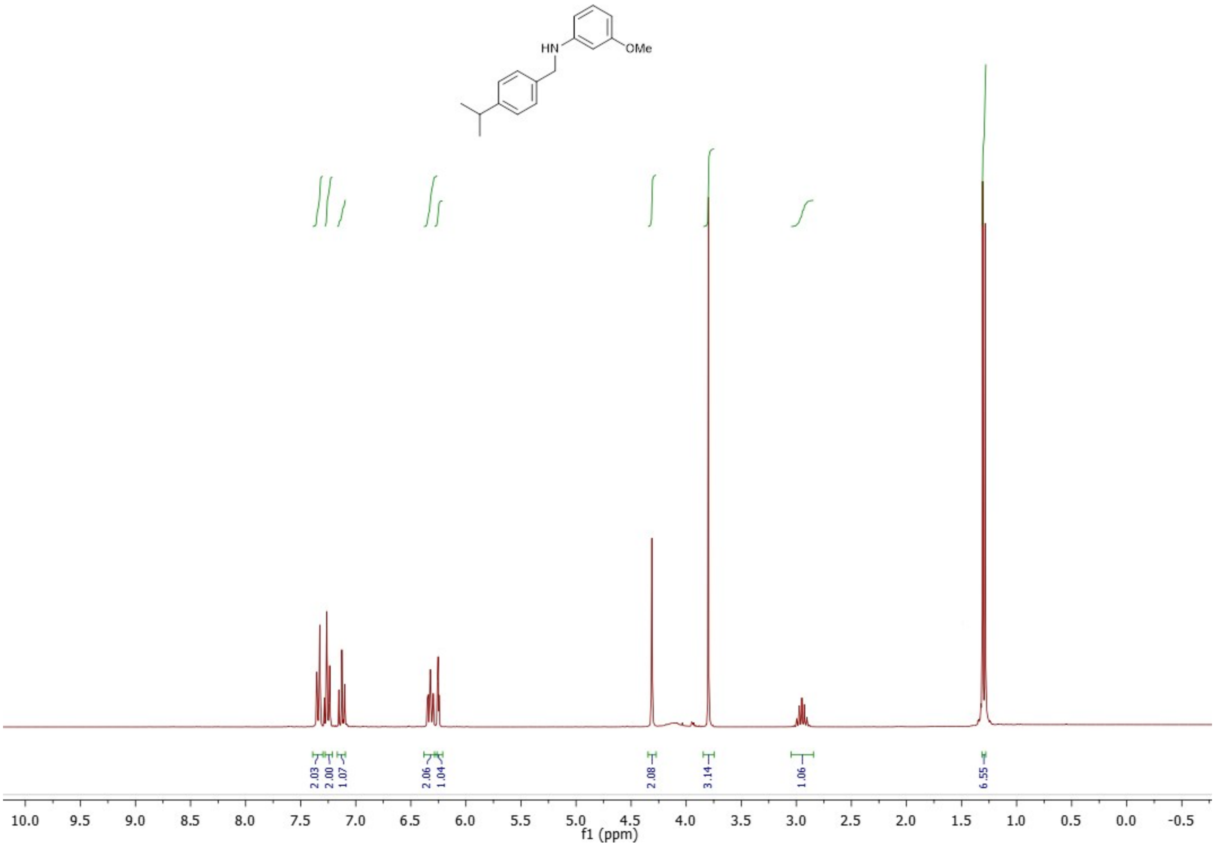

(^{13}C NMR, $CDCl_3$, 75 MHz)

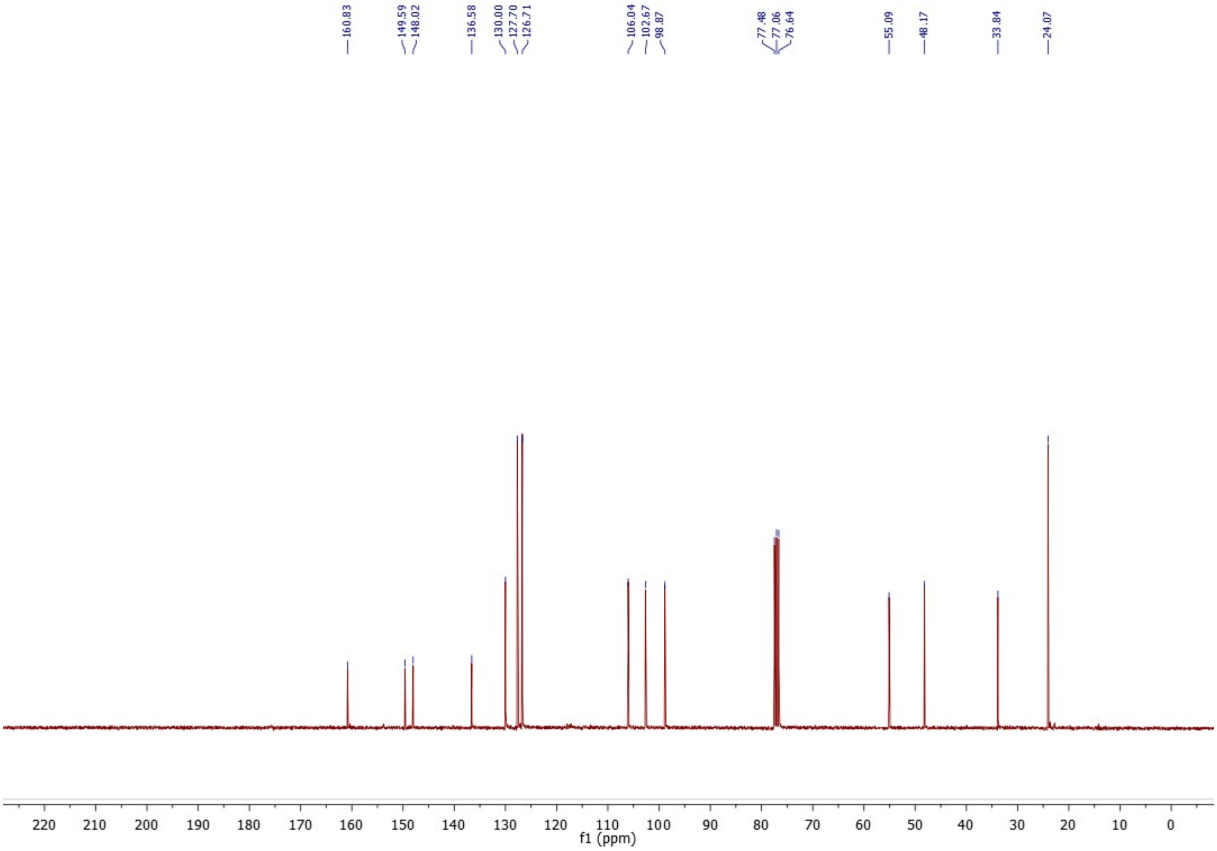

Methyl 3-((4-isopropylbenzyl)amino)benzoate (2p)

(^{1}H NMR, $CDCl_3$, 300 MHz)

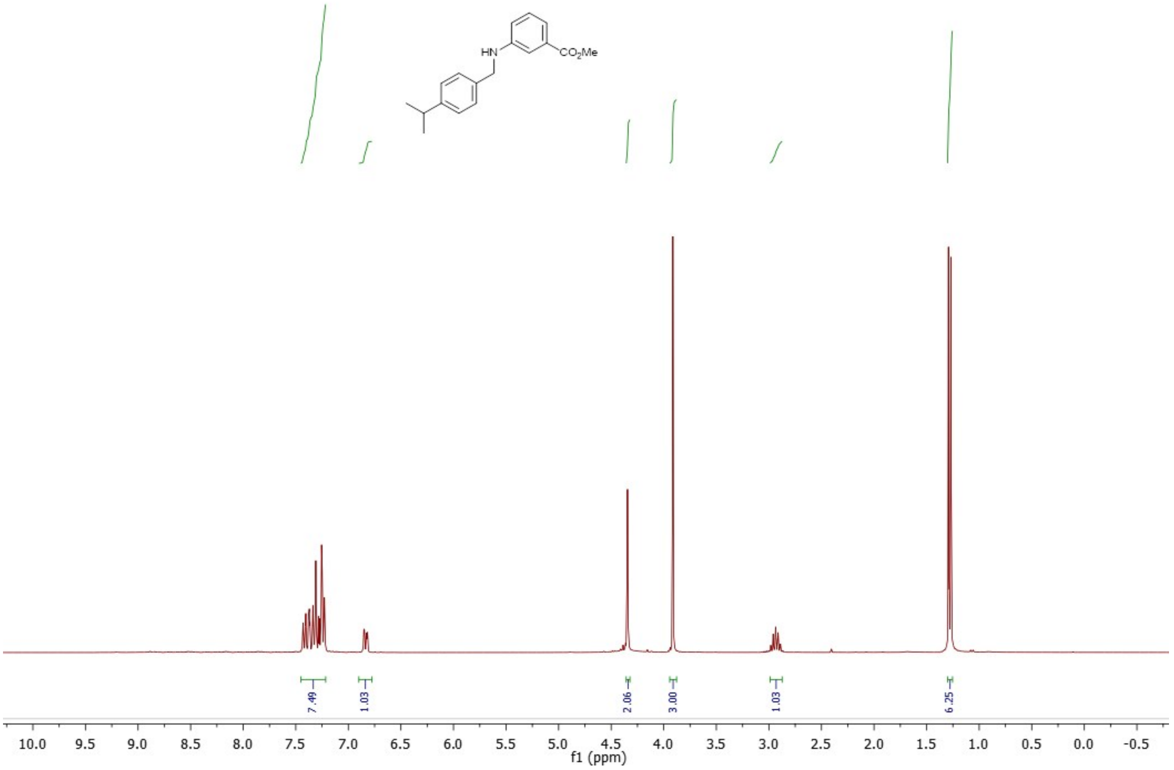

(^{13}C NMR, $CDCl_3$, 75 MHz)

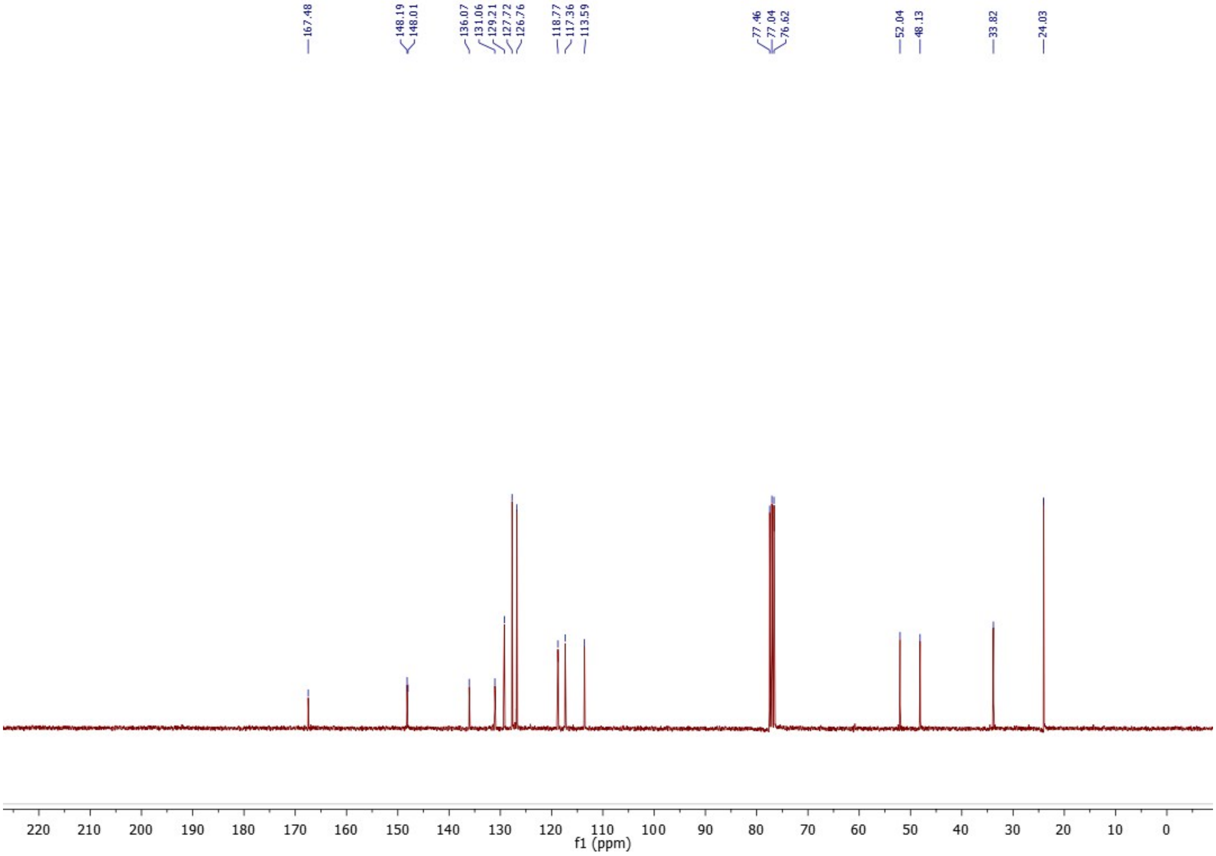

Supplement: Supplementary file 1 [file SC-010-C8SC04337J-s001.pdf]
